# Home Range and Habitat Selection of the Endangered Red Goshawk ( *Erythrotriorchis radiatus* ) Across Australia's Tropical Savannas

**DOI:** 10.1002/ece3.74042

**Published:** 2026-08-04

**Authors:** Christopher MacColl, Richard Seaton, Nicholas P. Leseberg, David A. Stewart, Stephen A. Murphy, James E. M. Watson

**Affiliations:** ^1^ School of the Environment University of Queensland St Lucia Queensland Australia; ^2^ Research and Recovery of Endangered Species Group University of Queensland St Lucia Queensland Australia; ^3^ Birdlife Australia Carlton Victoria Australia; ^4^ Queensland Department of the Environment, Tourism, Science, and Innovation Brisbane Queensland Australia; ^5^ Conservation Partners Malanda Queensland Australia

**Keywords:** AKDE, endangered, habitat selection, home range, raptor, red goshawk, resource selection functions

## Abstract

The Red Goshawk ( *Erythrotriorchis radiatus* ) is Australia's rarest and most threatened raptor, having disappeared from approximately two‐thirds of its breeding range and now persists across northern Australian tropical savannas. Despite this decline, its spatial requirements and habitat selections remain poorly understood within this environment, particularly in relation to anthropogenic disturbance. We conducted a long‐term movement study across northern Australia (2015–2022), tagging adults and juveniles (*n* = 23 individuals; 67,142 locations). Home range size and habitat selection were analysed for 13 range‐resident individuals using weighted autocorrelated kernel density estimators (wAKDE) and Resource Selection Functions (RSFs). Environmental covariates included both static (e.g., elevation, distance to water) and annually changing variables (e.g., habitat clearing, fire regime). Red Goshawks occupied extensive overall home ranges (mean 95% wAKDE = 601 ± 454 km^2^), with non‐breeders requiring substantially larger areas (1229 ± 496 km^2^) than breeders (412 ± 221 km^2^). Breeding adults relied on well‐defined core areas around nest sites (mean 50% wAKDE = 44.5 ± 27.1 km^2^). Habitat selection varied by breeding habitat: woodland breeders strongly select *Eucalyptus tetrodonta* woodlands relatively further from water, whereas riparian breeders avoid these woodlands, selecting long‐unburnt vegetation closer to water; although riparian sample sizes were limited. On Cape York Peninsula, individuals tracked during active clearing of woodlands for mining largely avoided cleared and restored areas. Collectively, our findings demonstrate the extensive spatial requirements of Red Goshawks and provide preliminary evidence of ecological niche differentiation between breeding habitat types within tropical savannas, although further data are needed to fully resolve this. Avoidance of cleared and restored areas indicates limited short‐term value of habitat restoration and provides a plausible mechanism linking habitat loss to the species' historical decline across its south‐eastern range. Intact woodlands and riparian gallery forests emerge as key habitats supporting extant breeding populations.

## Graphical abstract

We used long‐term GPS tracking to quantify home‐range size and habitat selection of the endangered Red Goshawk across tropical savannas of northern Australia. Red Goshawks occupied extensive overall home ranges, with well‐defined core ranges centred on nest sites. Habitat selection varied by breeding habitat type, indicating potential niche separation between woodland and riparian habitats. Avoidance of cleared and restored areas indicates limited short‐term value of habitat restoration and provides a plausible mechanism linking habitat loss to the species' historical decline across its south‐eastern range.

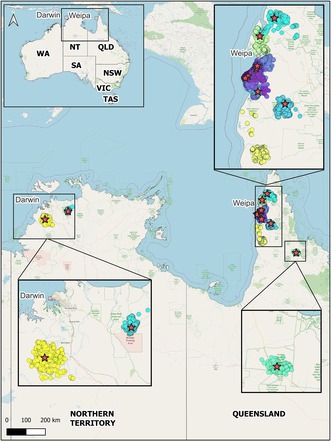

## 1 Introduction

Understanding species' home ranges and habitat selection is fundamental to effective conservation planning, as it reveals the spatial and ecological requirements necessary for survival and reproduction (Kelley et al. 2011; Choi et al. 2021). This information underpins decisions relating to habitat protection and restoration (Carey et al. 1990; McKellar and Clements 2023), infrastructure mitigation (Plumb et al. 2019; Murgatroyd et al. 2021), and the identification of areas suitable for reintroduction or recolonisation efforts (Mladenoff et al. 1995; D'Elia et al. 2015). Australia's Red Goshawk ( *Erythrotriorchis radiatus* ) is an endangered species of high conservation concern for which this information remains largely unavailable, primarily due to its rarity and cryptic behaviour (MacColl et al. 2023). However, recent advances in GPS tracking technology are now providing unprecedented opportunities to examine the spatial ecology of wide‐ranging species in fine detail (Hebblewhite and Haydon 2010; Kays et al. 2015).

The Red Goshawk is the rarest and most threatened raptor in Australia (Slater 1978; MacColl et al. 2023). Its breeding range has contracted by as much as two‐thirds since the 1970s, and breeding populations are now restricted to the tropical savannas of northern Australia—from Cape York Peninsula in the east to the Kimberley in the west (MacColl et al. 2023). Current estimates place the remaining population at approximately 670 breeding pairs (MacColl et al. 2021). Despite this, understanding of how the species utilises these landscapes remains limited, with only preliminary radio tracking of a few individuals (Aumann and Baker‐Gabb 1991) and some habitat assessments at nest sites (Czechura et al. 2011) available to infer its spatial requirements.

These shortfalls in specific autecological knowledge of Red Goshawk are compounded by the growing pressures on northern Australian tropical savanna ecosystems, including emerging threats from climate change (Petheram et al. 2012; Canadell et al. 2021), altered fire regimes (Bowman et al. 2022; Santos et al. 2022), invasive species (Setterfield et al. 2010) and habitat modification from mining and agricultural expansion (Williams et al. 2022). These processes have the potential to significantly alter the key habitat features, prey availability and nesting resources which Red Goshawks rely upon, yet the species' response to such landscape‐scale disturbances remains unknown.

To address these knowledge gaps, we conducted a multi‐year GPS–Argos tracking study of Red Goshawks across northern Australia. We examined home range size and its annual dynamics, including how it varies by age, sex, reproductive status, the number of young raised and breeding habitat type (woodland vs. riparian). We also modelled habitat selection including response to disturbance regimes such as annual fire and habitat clearing activities. As a unique case study, we tracked the annual expansion of clearing and restoration footprints around the bauxite mining landscapes of Weipa, Queensland and tested its effects on habitat selection by resident birds whose home ranges overlapped with these disturbances. Together, these analyses provide the first detailed insights into the species' spatial ecology, including identifying important habitat features and quantifying how anthropogenic disturbances affect space‐use. This information greatly improves our understanding of Red Goshawks' spatial requirements and provides an evidence base for conservation actions aimed at supporting their recovery and improving understanding of their historical decline.

## 2 Methods

### 2.1 Study Species

The Red Goshawk is a taxonomically distinct raptor (*Erythrotriorchis* sp.) endemic to the tropics and sub‐tropics of eastern and northern Australia. It is a bird‐eating specialist and the largest among the resident ‘hawks’ (*Tachyspiza* sp.) of Australia. It also exhibits among the highest degrees of reverse‐sexual size‐dimorphism of any raptor in the world (Baker‐Gabb 1984; MacColl et al. 2024). Red Goshawks occupy tall woodlands and riparian habitats in the tropical savannas, which it relies on for nesting and foraging opportunities (Marchant and Higgins 1993). It is an inherently rare species, with only very low population densities even in areas of apparently high habitat suitability (Aumann and Baker‐Gabb 1991). The breeding season is pronounced, spanning April (courtship) to January (fledgling dispersal), with 1–2 chicks produced annually (Aumann and Baker‐Gabb 1991).

### 2.2 Study Area

This study was conducted across the tropical savannas of northern Australia, specifically within the Cape York Peninsula region in Queensland, and the Top End region in the Northern Territory. The prevailing habitat consists of extensive tropical woodlands and savannas with grassy understoreys, interspersed with freshwater tributaries and wetlands. This region experiences a strong seasonal climate, characterised by heavy rainfall during the wet season (December–March), followed by a prolonged dry season (April–November). This strong seasonality drives an acute cycle of vegetation growth and senescence, resulting in highly combustible grassy fuel loads during the dry months. Consequently, these savannas are among the most fire‐prone ecosystems in the world (Dwyer et al. 2000).

Within this environment, Red Goshawks nest in semi‐open woodlands that retain tall, old‐growth vegetation with an open limb structure. Almost all verified nests (*n* = 66 individual nests representing 54 unique breeding territories from 2014 to 2025) recorded during this study were found in one of two distinct habitat associations: (i) Darwin stringybark (*Eucalyptus tetrodonta*) dominant woodlands occurring on ridges and low rises (*n* = 48); or (ii) along freshwater tributaries supporting mature stands of Weeping paperbark ( *Melaleuca leucadendra* ) gallery forest (*n* = 17), often associated with variable terrain, such as plateaus and gorges. Only one nest was recorded outside these two habitats. It occurred in a broad‐leaf, semi‐deciduous tree adjacent to water, was active only for a single season, and the breeding attempt failed. Therefore, based on this single record, we do not consider this vegetation type to be an important breeding habitat for the species.

Although our study area encompassed two Australian states, most movement data were collected on western Cape York Peninsula, particularly around the townships of Aurukun and Weipa, Queensland. This region includes extensive mining‐affected landscapes associated with bauxite extraction, which involves the removal of vegetation and topsoil to access deposits below.

### 2.3 Data Collection and Filtering

#### 2.3.1 Capture and Tagging

The aim of this study was to collect fine‐scale movement data that would allow us to draw robust conclusions about the Red Goshawk's spatial requirements. To achieve this, we deployed solar‐powered satellite transmitters on both adult and juvenile birds to track their movements over time. Red Goshawks were captured near nest sites using a remotely triggered 8‐ft bow‐net (Northwoods Falconry, Olympia, WA, USA) and a live lure bird. Adults were trapped during the breeding season—females during the mid‐late nestling phase (Sep–Oct) and the sole adult male during nest building (Jun–Jul) when nest attendance is highest (Debus et al. 2015). Juveniles were captured during the post‐fledging period (Nov–Jan), once fully grown and capable of flight and hunting attempts. Juvenile males, being smaller, developed more rapidly than juvenile females and were therefore often tagged earlier.

Tags were attached using a backpack‐style harness made of Teflon or Spectra ribbon (Bally Ribbon Mills, Bally, PA, USA), with biodegradable cotton stitching designed to break down over time and act as a weak link on the breast (Bierregaard 2014). The harness looped under the wings and over the shoulders, with the tag positioned just below the patagium along the upper back. Harness fit was repeatedly checked by two experienced observers and secured using dental floss and super glue.

Two types of solar‐powered satellite transmitters were deployed in this study: 17 g GPS‐Argos and 9.5 g Argos‐only Platform Terminal Transmitters (PTTs) (Microwave Telemetry Inc., Columbia, MD, USA). Both transmitters relayed data remotely via the Argos satellite system (Collecte Localisation Satellites, Toulouse, France), with incoming location data automatically parsed and stored in a standardised format on *Movebank* (https://www.movebank.org; Study ID: 2076371267), a global repository for animal movement data.

Due to the species' pronounced sexual size dimorphism (Aumann and Baker‐Gabb 1991), the smaller Argos PTTs were generally fitted to males, while the larger GPS‐Argos tags were better suited to females. This approach is consistent with commonly applied guidelines to minimise tag loads relative to body mass (e.g., Kenward 2001). Tag deployment occasionally deviated from this protocol depending on tag availability at the time of deployment; however, tag loads were always within permitted limits (males: mean ± SD = 2.47% ± 0.99% of body mass; females: 1.53% ± 0.33% of body mass).

#### 2.3.2 Movement Data

GPS‐Argos transmitters were programmed to acquire fixes at 3‐h intervals during daylight (i.e., 06:00, 09:00, 12:00, 15:00, 18:00), while Argos‐only PTTs were programmed on an 8 h ON/40 h OFF duty cycle. However, the Argos PTTs were equipped with a secondary programming option that allowed transmission outside scheduled ON periods provided battery charge was sufficient.

Data resolution varied between tag types. GPS fixes provided high spatial accuracy (±18 m), while Argos‐based fixes depended on the number of satellite messages received and were derived from Doppler shift calculations. Argos data were assigned to location classes (LC) ranging in estimated error from > 1500 m (LC0) to < 250 m (LC3). To ensure spatial reliability, only the most accurate Argos location classes—LC1 (< 1500 m), LC2 (< 500 m) and LC3 (< 250 m)—were retained for analysis.

#### 2.3.3 Tag Deployment and Animal Selection

**FIGURE 1:**
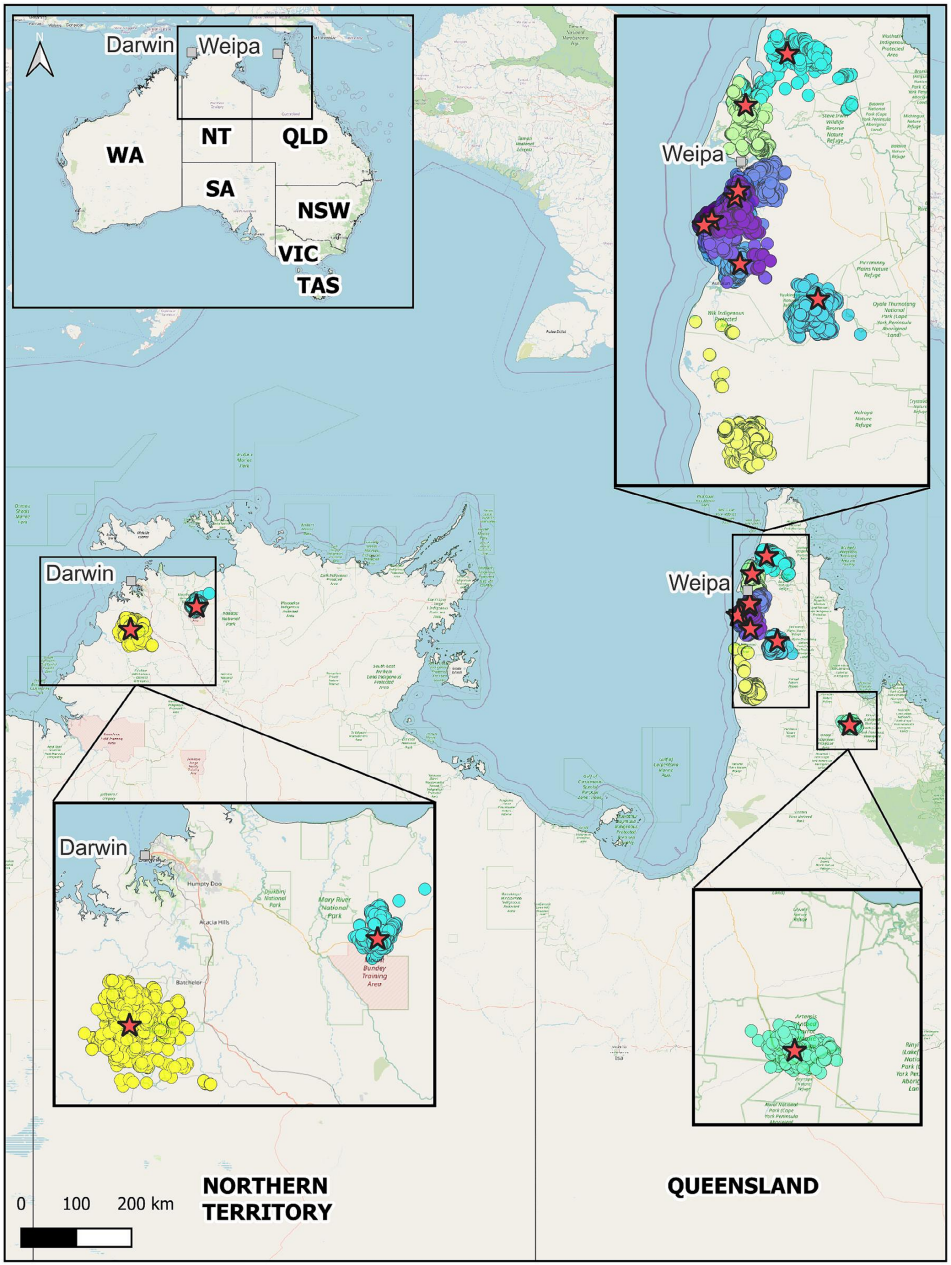
Movement data for range‐resident Red Goshawks included in home range and habitat selection analysis (*n* = 13). Coloured circles represent GPS locations for individual birds, and red stars indicate nest locations within active breeding territories.

A total of 23 Red Goshawks were tagged between 2015 and 2022 for this study. Details of all individuals tagged including age, sex, habitat, tag type, number of location fixes and duration of tracking period are provided in Table 1, and the movement data of individuals used in home range and habitat selection analysis are shown in Figure 1.

**TABLE 1:** Summary of all tagged Red Goshawks including tracking duration and number of location fixes.

Tag ID	Age at tagging	Sex	Breeding habitat^a^	Tag type	Date tagged	Tracking duration (Days)^b^	# of locations
150,622^c^	Adult	Female	Woodland	GPS	26‐September‐2015	93	630
150,621^c^	Juvenile	Female	Woodland	GPS	12‐January‐2016	561	3098
157,650^c^, ^d^	Adult	Female	Woodland	GPS	12‐September‐2018	875	4331
157,651^c^	Adult	Female	Woodland	GPS	20‐September‐2018	181	900
37740_1	Juvenile	Female	Woodland	GPS	06‐December‐2018	133	662
66,559^c^, ^d^	Adult	Female	Riparian	GPS	20‐October‐2019	1887^e^	9008^e^
66,560^c^, ^d^	Juvenile	Female	Woodland	GPS	17‐December‐2019	1691	8404
66,561	Juvenile	Female	Woodland	GPS	20‐December‐2019	372	1841
37,737^c^	Adult	Female	Woodland	GPS	15‐October‐2020	831	4091
37740_2^c^	Adult	Female	Woodland	GPS	21‐October‐2020	1420	7053
202,347	Juvenile	Male	Riparian	GSP	14‐December‐2020	39	192
66,564_1	Juvenile	Male	Woodland	GSP	18‐December‐2020	45	225
202,348^c^	Adult	Female	Woodland	GPS	14‐October‐2021	1027	5091
202,346^c^	Adult	Female	Woodland	GPS	16‐October‐2021	1180	5824
220,633	Juvenile	Female	Woodland	GPS	02‐December‐2021	400	2007
66,564_2^c^	Juvenile	Female	Woodland	GPS	05‐December‐2021	1119	5508
220,636^c^	Juvenile	Female	Woodland	Argos	06‐December‐2021	773^e^	4398^e^
220,634	Juvenile	Female	Woodland	Argos	07‐December‐2021	110	865
226,138^c^	Adult	Male	Woodland	Argos	29‐June‐2022	171	1150
226,137	Juvenile	Female	Woodland	GPS	02‐December‐2022	41	208
237,146	Juvenile	Male	Woodland	Argos	03‐December‐2022	134	815
220,635	Juvenile	Male	Woodland	Argos	09‐December‐2022	13	65
237,147	Juvenile	Male	Woodland	Argos	12‐December‐2022	142	776

*Note:* Individuals are presented in chronological order with those selected for home range and habitat selection analysis indicated.

^a^ Woodland = *E. tetrodonta* woodlands; Riparian = *M. leucadendra* gallery forest along freshwater creeks.

^b^ Sampling duration for Argos tags includes only number of days in which data was transmitted (i.e., the ON days) not the total duration the tag was worn by the individual.

^c^ Individuals used in wAKDE home range and RSF habitat selection analysis.

^d^ Migratory individuals whose wAKDE and habitat selection analysis was restricted to the breeding home range only.

^e^ Still receiving location data at the time of analysis.

In total, nine adults and 14 juveniles were tagged, although three juveniles later established territories and commenced breeding during the tracking period. Sex ratios were skewed toward females, with 18 females and five males tagged overall, including only one adult male. Breeding habitat was also unevenly represented, with most individuals occupying woodland habitats (*n* = 21) and only two individuals—including one adult—occupying riparian habitats.

These discrepancies reflect logistical constraints and emerging knowledge while studying this species, rather than deliberate sampling bias. Discoveries made during the course of this study alerted us to the possibility of two distinct breeding habitats, which we could not fully investigate within the constraints of this project. Additionally, trapping adult males required different approaches to those used successfully on adult females and juveniles of either sex, and was only achieved later in the study. As a result, the dataset is unevenly distributed across sex and habitat types, which should be considered when interpreting results.

Only individuals meeting the requirements for range residency were selected for home range estimation using weighted Autocorrelated Kernel Density Estimators (wAKDE) (Fleming et al. 2019) and for habitat selection modelling using Resource Selection Functions (RSFs) (Alston et al. 2023). Two filtering methods were required to ensure these conditions were met and our dataset utilised the maximum possible number of individuals:

1. *Juvenile data*: Birds tagged as juveniles and which subsequently survived into later years and life stages had their first year of data removed (*n* = 3), as juveniles exhibited wide‐ranging, exploratory movements in their first year but appeared to establish range residency from their second year onwards. The retained data included both non‐breeding and breeding life stages, with age at first breeding typically occurring in their second or third years.
2. *Migratory data*: A subset of adults (*n* = 3, ~23% of adults tagged) displayed seasonal migratory behaviour, moving between distinct breeding (~April–December) and non‐breeding (~January–March) home ranges. For these birds, we removed non‐breeding season data so that our analysis focused on their breeding home range only, making it comparable with all other individuals who were year‐round residents on their breeding territories.

Overall, the total movement dataset comprised 67,142 location fixes across 13,238 tracking days. Of these, 59,073 locations were obtained from GPS and 8069 estimated using Argos Doppler. Tracking durations ranged from 14 to 1907 days (median = 370; mean ± SD = 600 ± 587 days), with the longest‐tracked individual still transmitting data at the time of analysis.

### 2.4 Statistical Analysis

#### 2.4.1 Auto‐Correlated Kernel Density Estimators (AKDE)

To estimate Red Goshawk home ranges, we applied weighted auto‐correlated kernel density estimators (wAKDE) using the *ctmm* package in *R* (Calabrese et al. 2016; R Core Team 2021). wAKDE offers several advantages over conventional home range estimators by accounting for spatial auto‐correlation, small sample size and irregular sampling schedules (Fleming et al. 2019; Silva et al. 2022). These factors can otherwise lead to over or under‐estimation of home range size (Noonan et al. 2019).

Home range estimation followed four main steps: First, assessing whether individuals were range resident via semi‐variogram analysis (Fleming et al. 2014). Second, selecting the best‐fit continuous‐time movement model for each individual (Calabrese et al. 2016). Third, estimating utilisation distributions and delineating 95% and 50% home range contours using area‐corrected AKDE (Fleming and Calabrese 2017). Fourth, applying corrections for residual biases such as irregular sampling or low effective sample sizes using weighting or bootstrapping techniques (Fleming et al. 2019; Silva et al. 2022).

**FIGURE 2:**
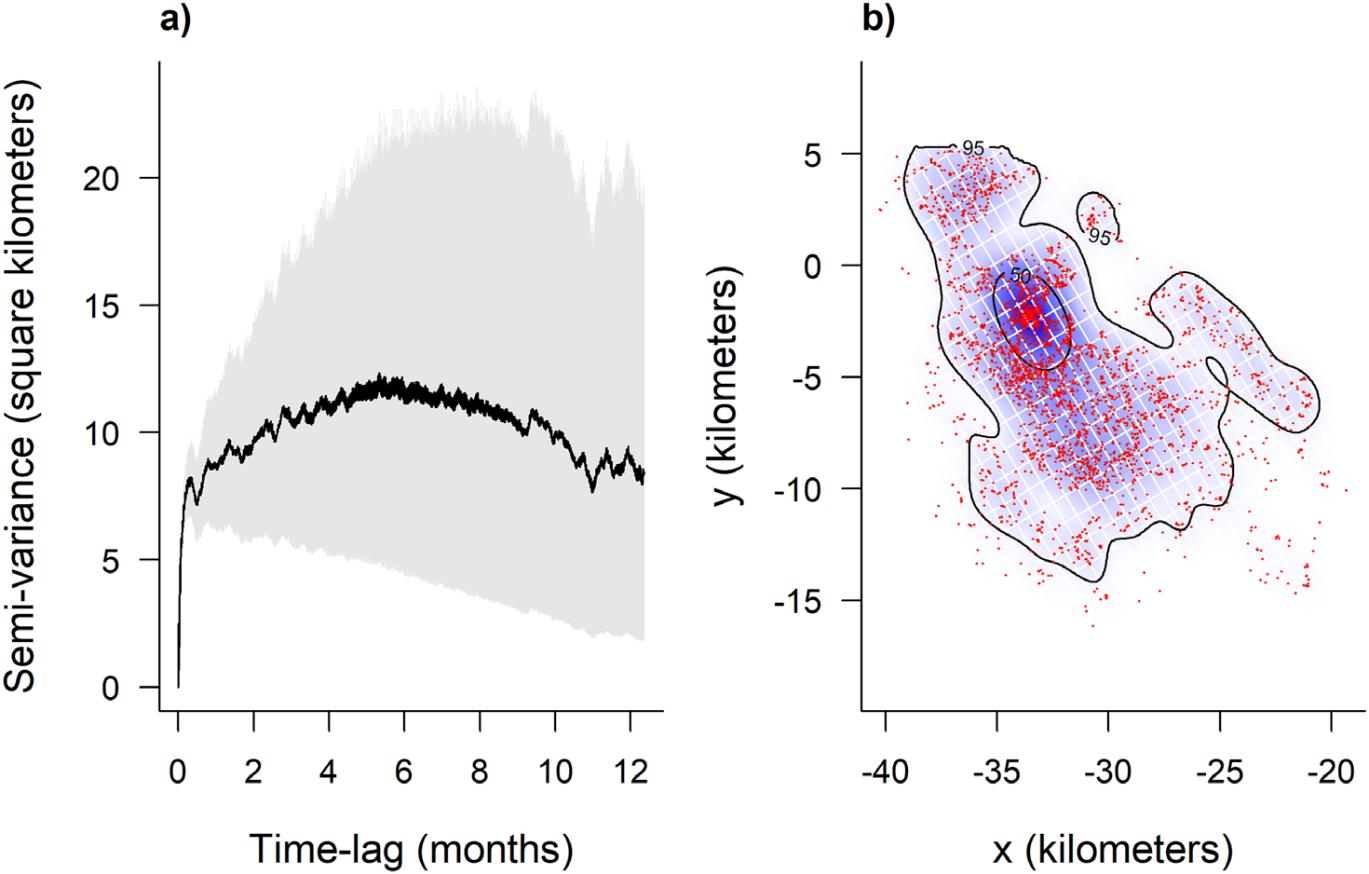
Semi‐variogram and utilisation distribution for a representative tracked Red Goshawk individual (#202346) used in home range estimation. (a) semi‐variogram used to assess range residency and model fit, and (b) the corresponding utilisation distribution, including 95% and 50% isopleths.

While most individuals exhibited semi‐variograms that reached a clear plateau consistent with range residency (Figure 2), a small number showed less well‐defined asymptotes (see Data S1). These cases were assessed individually, and only individuals considered to meet the assumptions of range residency were retained for home range estimation (*n* = 13; see Table 1).

Home range sizes were calculated for each individual (cumulative and annual) using 95% and 50% weighted AKDE (wAKDE) isopleths, expressed in square kilometres. Cumulative home range estimates were then pooled and averaged across tracked individuals by sex, breeding habitat (woodland vs. riparian) and breeding status (breeder vs. non‐breeder). Annual home ranges were compared by age (between 1 and 4 years old for known‐age individuals tagged as juveniles) and brood size of breeders (i.e., number of fledglings raised each year).

Details of wAKDE model selection, absolute sample size (number of location fixes) and effective sample size (degrees of freedom) for each individual are provided in Data S2. All wAKDE estimates utilised weighting to account for irregular sampling between tag types (GPS vs. Argos‐only), and effective sample sizes were sufficient to ensure reliable estimation without additional bias corrections (e.g., bootstrapping).

### 2.5 Habitat Selection

#### 2.5.1 Environmental Covariates

**FIGURE 3:**
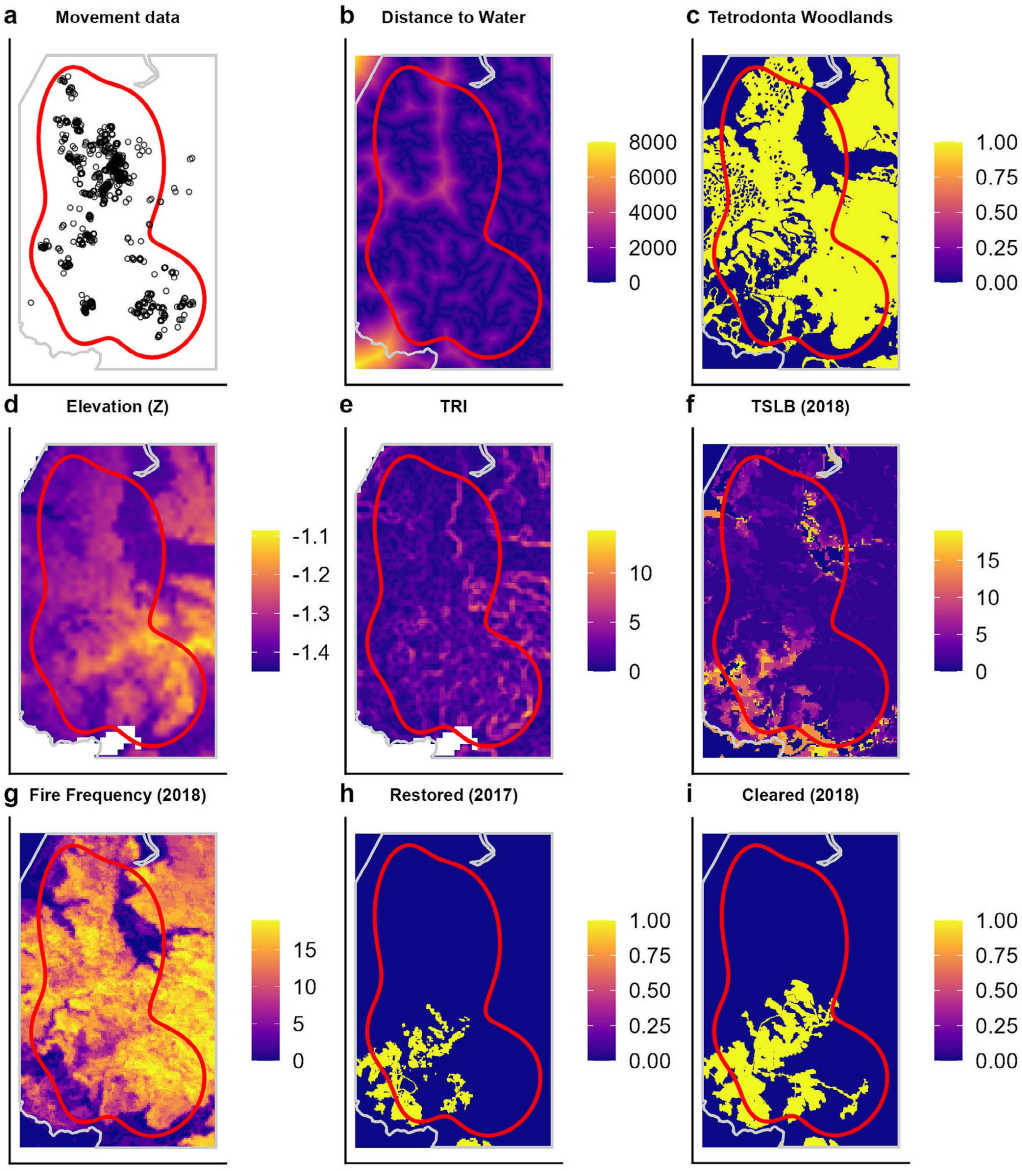
Example of environmental raster layers and movement data used in habitat selection modelling for an adult female (#157651) tracked in 2018 on western Cape York Peninsula. This individual was selected as a representative example of a range‐resident bird with access to all available environmental variables used in RSF analysis, including cleared and restored areas within mining landscapes. Panel (a) displays the individual's GPS movement data and 95% wAKDE home range contour (red polygon). Panels (b–i) show environmental raster layers cropped to the bounding box of the 95% wAKDE home range and buffered by 2 km to set the availability area. Used versus available locations were derived from within this extent for RSF modelling. Descriptions of individual variables are provided in Table 2.

**FIGURE 4:**
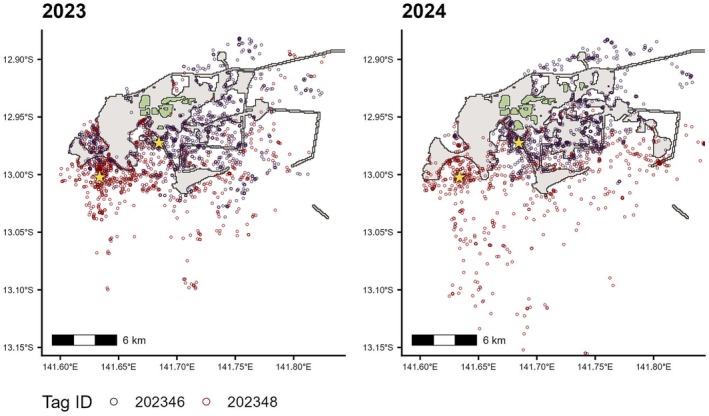
Annual expansion of habitat clearing and restoration on western Cape York Peninsula. Grey polygons show cleared areas and green polygons show restoration areas for calendar years 2023–2024. Hollow circles represent GPS locations for two tracked breeding females (#202346 & #202348) with yellow stars indicating their respective nest locations within their breeding territory. All remaining areas (white) represent intact habitat.

To assess habitat selection by Red Goshawks, we compiled a series of national and regional‐scale environmental raster layers considered relevant to the species' ecology (Table 2; Figure 3). These included static topographic variables, such as elevation and distance to water, as well as dynamic variables that changed annually, such as fire frequency and time since last burn (TSLB). In mining‐affected landscapes on western Cape York Peninsula near the township of Weipa, Queensland, we also incorporated cumulative clearing and restoration footprints to represent the ongoing removal of tropical woodlands for bauxite extraction, followed by subsequent attempts to restore these habitats post‐mining (Figure 4).

**TABLE 2:** Environmental rasters used in Resource Selection Function (RSF) modelling of habitat selection in Red Goshawks.

Raster layer	Temporal resolution	Source	Processing notes	Rationale
Elevation	Static	SRTM Digital Elevation Model (Geoscience Australia 2025a)	Standardised (*z*‐scores)	Nest locations associated with ridges and variable topography (Aumann and Baker‐Gabb 1991; Czechura et al. 2011)
Terrain Ruggedness Index (TRI)	Static	Derived from elevation	15 × 15 moving window	As above
Distance to water	Static	Surface Hydrology Lines (Regional) (Geoscience Australia 2025b)	Euclidean distance to nearest watercourse^a^	Nest locations associated with proximity to freshwater (Aumann and Baker‐Gabb 1991)
Tetrodonta Woodlands	Static	National Vegetation Information System (NVIS) v7.0 (DCCEEW 2020)	Binary raster (1 = tetrodonta woodland)^b^	Nest locations widely found in *E. tetrodonta* woodlands (Aumann and Baker‐Gabb 1991; Czechura et al. 2011)
Fire frequency	Annual	Long‐term fire frequency (North Australia and Rangelands Fire Information Service 2025)	Years burnt (2000–present), resampled to 100 m	Fire is a key ecological process in northern Australia (Russell‐Smith and Whitehead 2015; Edwards et al. 2021)
Time since last burn (TSLB)	Annual	Long‐term TSLB (North Australia and Rangelands Fire Information Service 2025)	Years since last burn, resampled to 100 m	As above
Habitat clearing	Annual	Landsat 8 & 9 OLI‐TIRS surface reflectance (Fuqin et al. 2019)	Cumulative clearing footprints (2014–2024), binary raster (1 = cleared)	Habitat loss is a known threat to Red Goshawks (Ward et al. 2019, 2022)
Restoration	Annual	Rio Tinto Weipa restoration vector layer	Cumulative restoration footprints (2014–2024), binary raster (1 = restored), lagged 1 year (*t* − 1)^c^	Restoration is tested as a binary covariable for selection within mining‐affected landscapes^d^

^a^ Hydrology lines were derived from a DEM, rasterised, and distance from each raster cell calculated using Euclidean distance.

^b^ MVG classes 3 & 5 broadly represent *E. tetrodonta* woodlands but are not exclusive.

^c^ Restoration lag applied to reflect delayed effect of seeding and planting in Nov–Dec each year.

^d^ Based on conditions present during each tracking year; differences in restoration age and associated structural variation were not explicitly assessed.

All environmental rasters were standardised to 100 m resolution and projected to EPSG:3577 (Albers Equal Area). Continuous layers were resampled using bilinear interpolation, and categorical layers using nearest‐neighbour.

#### 2.5.2 Resource Selection Function (RSF) Modelling

We applied Resource Selection Function (RSF) modelling to quantify habitat selection in Red Goshawks using the *ctmm* package in *R* (R Core Team 2021; Fleming and Calabrese 2023). RSFs estimate the relative probability of selection by comparing environmental characteristics at used locations (i.e., where an individual was tracked) with those at available locations (i.e., areas accessible within the home range but not used) (Manly et al. 2007).

Models were fit annually for each individual‐year (*n* = 38) from 13 range‐resident Red Goshawks (see Table 1). All environmental covariables used to test habitat selection were available to each individual, although sample sizes were more limited in those occupying riparian breeding habitats (*n* = 1 individual, 6 individual‐years) compared to woodlands (*n* = 12 individuals, 31 individual‐years). The only exception was a subset of individuals (*n* = 6) on western Cape York Peninsula whose home ranges were exposed to habitat clearing (*n* = 13 individual‐years) and restoration (*n* = 10 individual‐years) activities. Exact sample sizes for each covariate are provided in Table 4 alongside the RSF results.

To align telemetry and environmental data in time and space, annual raster layers were matched to the calendar year of movement data (e.g., clearing; see Figure 4), whereas static covariates remained constant over time (e.g., elevation, distance to water; see Figure 3). All environmental rasters were cropped to the bounding box of each individual's 95% wAKDE home range with an additional 2 km buffer applied to ensure adequate coverage of habitats available for selection (Alston et al. 2023). Raster values were extracted within each individual's availability area, and models fitted using a Montecarlo integration method to account for spatial autocorrelation in movement data (Alston et al. 2023). RSF model outputs were saved for each individual‐year to facilitate analysis of habitat selection at both the individual and group level.

We extracted mean coefficient estimates and 95% confidence intervals for each covariate from individual‐year models and summarised population patterns (means ± CIs). This hierarchical approach reduces potential bias associated with unequal sampling frequency (e.g., GPS vs. Argos‐only tags) or tracking duration among individuals, as selection is estimated relative to availability within each individual‐year home range rather than being pooled across the full dataset. Each environmental covariate was considered to significantly influence habitat selection when group‐level means and CIs did not overlap zero, with positive coefficients indicating selection for a covariate, while negative coefficients indicated avoidance. RSF results were categorised by breeding habitat type (woodland vs. riparian) to assess habitat‐related differences in selection and landscape context.

Finally, to assess whether group‐level selection patterns were disproportionately influenced by individual‐level variation, a sensitivity analysis was undertaken. The group‐level analysis was repeated after excluding any individual exhibiting markedly different habitat selection, and the resulting coefficient estimates and confidence intervals were compared with those obtained from the full dataset.

## 3 Results

### 3.1 Home Range

Red Goshawks occupied substantial overall home ranges, with mean home range size (95% wAKDE) estimated at 600.6 km^2^ (SD = 454.2 km^2^, 95% CI = 353.7–847.5) across all individuals. The mean core range area (50% wAKDE) was estimated at 98.6 km2 (SD = 116.4 km^2^, 95% CI = 35.3–161.9). However, home range size varied significantly by breeding status, with non‐breeders occupying much larger areas (1229 ± 496 km^2^) compared to breeders (411.9 ± 221 km^2^; Table 3). Breeding males appeared to occupy smaller home ranges (202.2 km^2^) compared to breeding females (435.2 km^2^ ± 221.0), although this estimate was based on just a single individual male. The single range‐resident riparian breeder tracked during the study also maintained a larger home range (665.0 km^2^) than woodland breeders (383.8 ± 214.6 km^2^), although this estimate was based on one individual.

**TABLE 3:** Mean cumulative (overall) home range size (50% and 95% wAKDE) by sex, breeding status and habitat.

Group	*n*	Mean core range (50% wAKDE, km^2^)	SD	95% CI	Mean home range (95% wAKDE, km^2^)	SD	95% CI
All breeders	10	44.5	27.1	27.7–61.3	411.9	221.0	274.9–548.9
Breeding male	1	42.5	—	—	202.2	—	—
Breeding female	9	44.8	28.7	26.0–63.5	435.2	221.0	290.8–579.6
Non‐breeders	3	286.2	121.2		1229.5	496.4	667.8–1791.2
Woodland breeders	9	44.5	28.8	25.8–63.3	383.8	214.6	243.6–524.0
Riparian breeders	1	44.4	—	—	665.0	—	—

**FIGURE 5:**
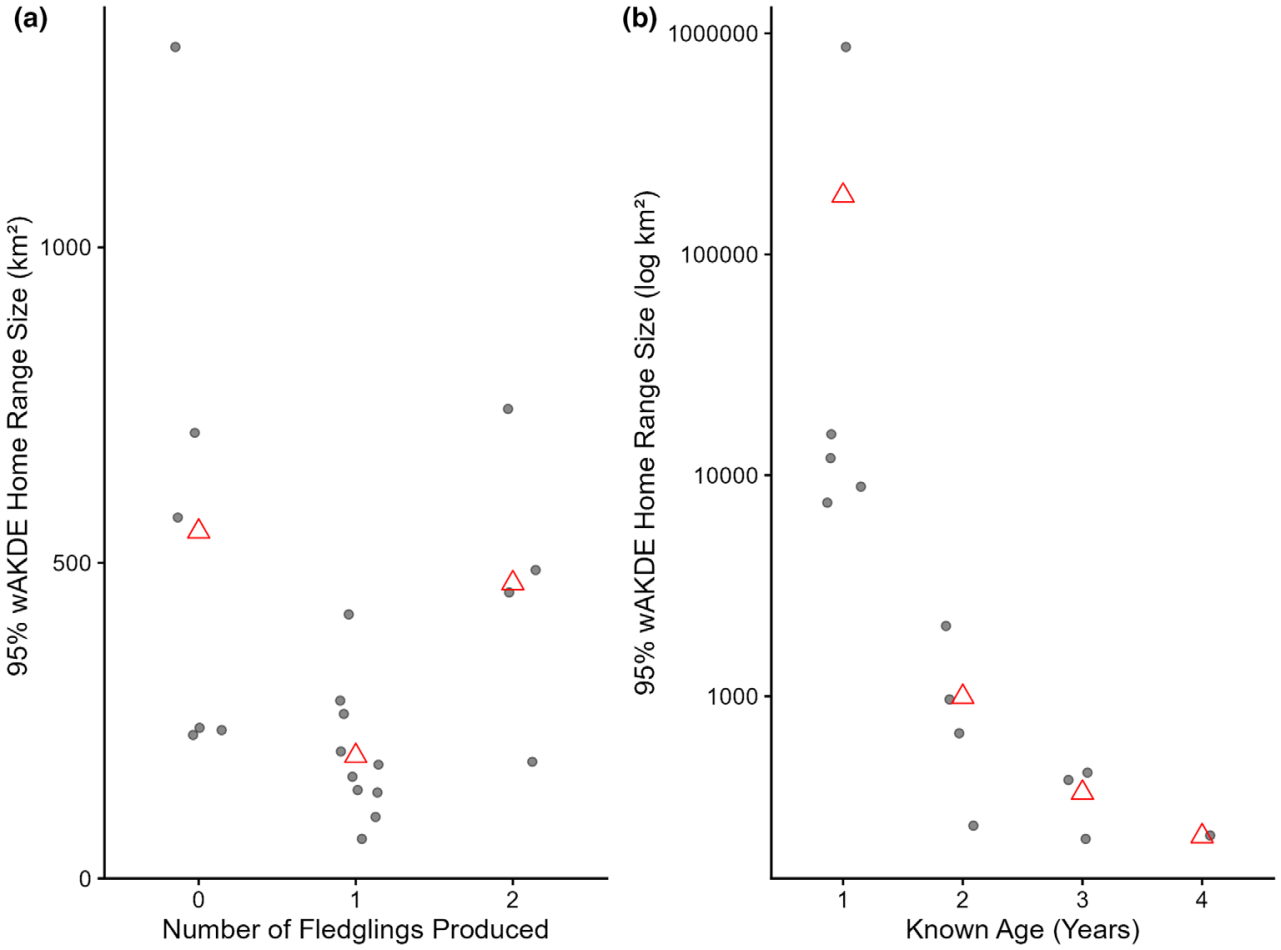
Annual home range size (95% wAKDE) in relation to (a) brood size and (b) age. Brood size is measured as the number of fledglings produced per breeding season, and age represents the known age of individuals tagged as juveniles. The y‐axis is log‐transformed in panel (b). Mean values are represented by red triangles.

Annual home range size was influenced by age and brood size (Figure 5; Data S3). Individuals producing two fledglings (*n* = 4) ranged wider than those producing one (*n* = 10) (Figure 5a). In contrast, individuals that failed to produce any fledglings in their breeding attempts occupied even larger home ranges on average (*n* = 6), although these values exhibited greater variability. Annual home range also declined with age, as individuals progressively required less space each year from their first year onwards (Figure 5b).

### 3.2 Habitat Selection

#### 3.2.1 Individual‐Level Habitat Selection Patterns

**FIGURE 6:**
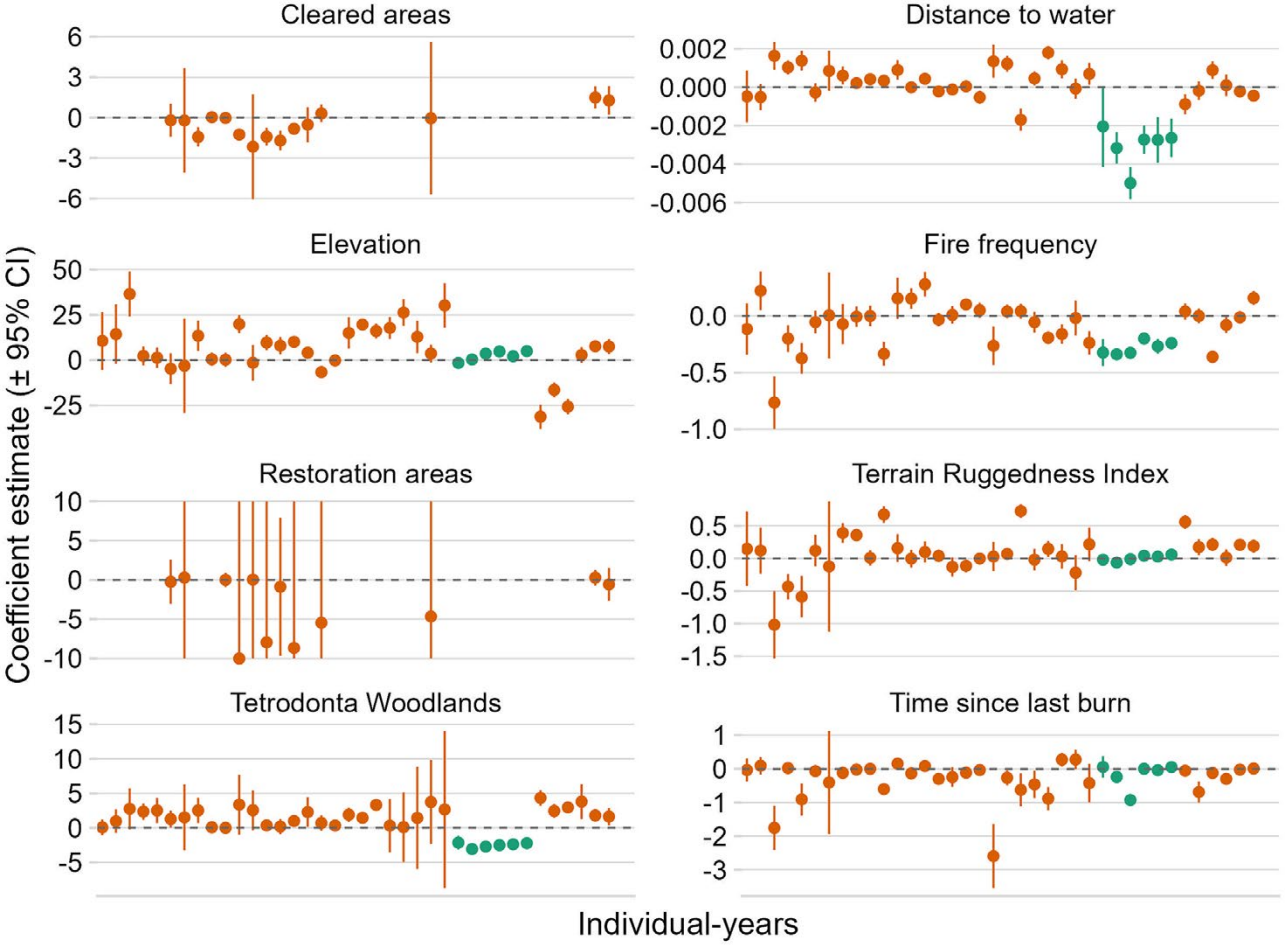
Individual‐level RSF coefficient estimates (mean ± 95% CI) by breeding habitat type (woodland vs. riparian). Woodland estimates are shown in orange and riparian estimates in green for each environmental covariate used to test habitat selection. Individual‐year estimates on the x‐axis represent the tag ID and tracking year for each individual.

RSFs were fit to 38 individual‐years from 13 range‐resident Red Goshawks between 2015 and 2024 (see Data S4). Individual‐level variability was evident across most environmental predictors, with differences in both the strength and direction of selection patterns among individuals (Figure 6). Despite this variation, some consistent patterns emerged, particularly when results were categorised by breeding habitat (woodland and riparian).

Notably, individual‐year RSFs revealed that a single individual (#66564) exhibited selection for cleared areas, whereas all other individuals for whom these habitats were available showed either strong avoidance or no response. Closer inspection of the movement data indicated that #66564 primarily used forest edges rather than extensively cleared zones, and that these selections took place in 2023 and 2024 when this bird was just 2 and 3 years old, respectively.

**FIGURE 7:**
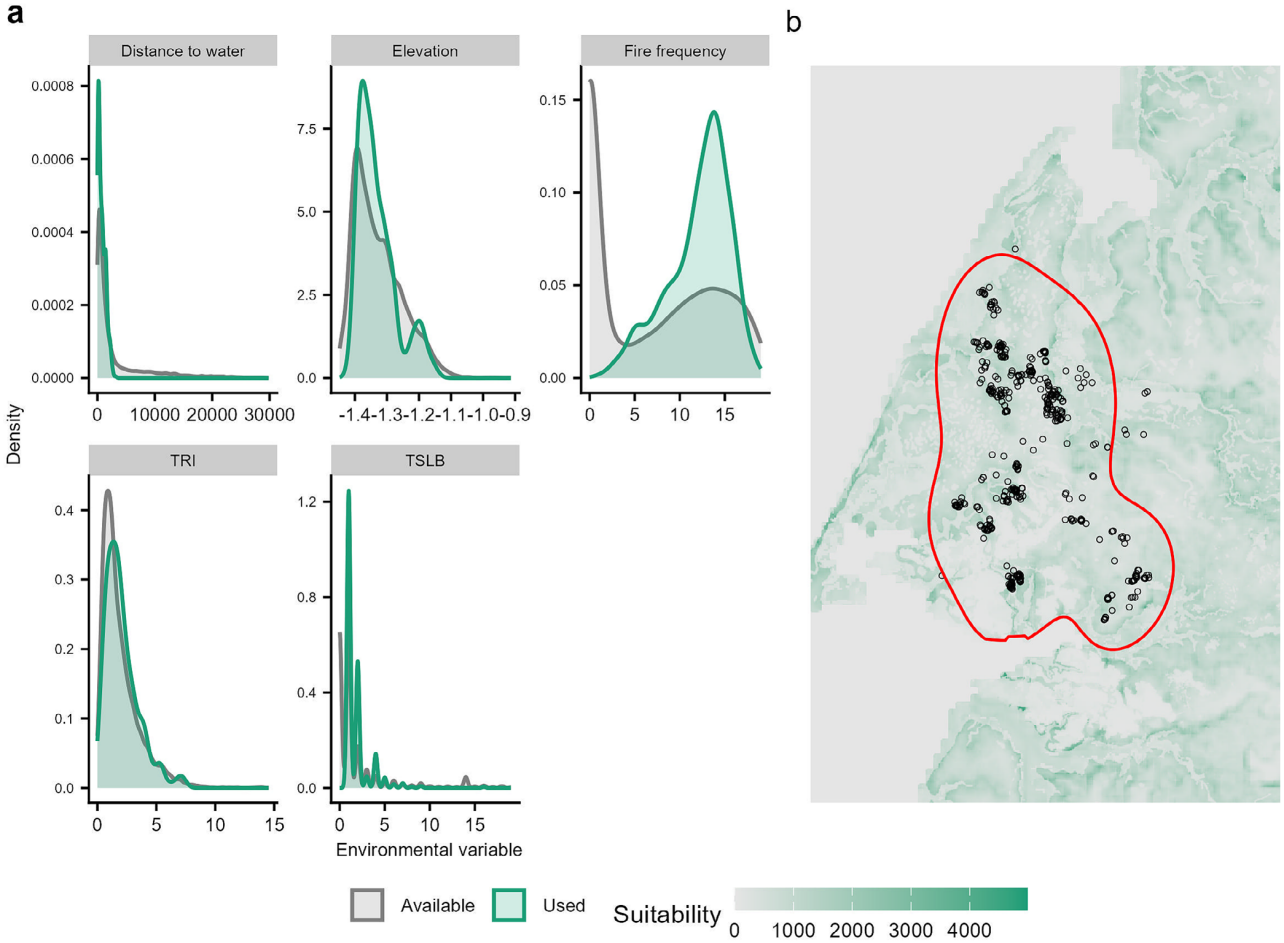
Habitat selection and predicted suitability for a representative Red Goshawk individual in 2018 (#157651; as shown in Figure 3), including (a) distributions of continuous environmental covariates at used and available locations, and (b) the predicted habitat suitability surface with movement locations (circles) and the 95% wAKDE home range contour (red polygon). Binary environmental covariates (e.g., clearing and Tetrodonta woodlands) are not shown as their distributions are not scalable (i.e., values are 0 or 1).

These individual‐level selection patterns were reflected in the spatial distribution of predicted habitat suitability within each individual's home range (Figure 7a). Interannual variability in selection may reflect changes to local environmental conditions, particularly for dynamic variables that change annually (e.g., TSLB and fire frequency), given that RSF models were fitted on an individual‐year basis. These interannual differences were also reflected in the corresponding distributions of used and available locations employed in the modelling process within each individual home range (Figure 7b).

#### 3.2.2 Group‐Level Selection Patterns and Variation by Breeding Habitat

Across all individuals, Red Goshawks consistently selected areas of relatively higher elevation (mean *β* = 5.659, 95% CI = 1.469 to 9.849; see Data S5) but otherwise showed distinct differences in habitat selection based on their breeding habitat type (Table 4).

**TABLE 4:** Group‐level RSF coefficient estimates (mean ± 95% CI) categorised by breeding habitat (woodland vs. riparian).

Covariate	Habitat	*n*	Mean estimate	SD	95% CI lower	95% CI upper	Interpretation
Cleared areas	Woodland	6 ind.; 15 ind‐yrs	−0.446	1.041	−0.973	0.081	No effect
Restoration areas	Woodland	6 ind.; 12 ind‐yrs	−3.233^b^	1.188	−5.562	−0.904	Avoidance
Distance to water	Riparian	1 ind.; 6 ind‐yrs	−0.003^b^	0.001	−0.004	−0.002	Selection for areas closer to water
	Woodland	12 ind.; 32 ind‐yrs	0.000^b^	0.001	0.000	0.001	Selection for areas further from water
Tetrodonta Woodlands	Riparian	1 ind.; 6 ind‐yrs	−2.500^b^	0.344	−2.775	−2.224	Avoidance
	Woodland	12 ind.; 32 ind‐yrs	1.770^b^	1.253	1.336	2.204	Selection
TRI^a^	Riparian	1 ind.; 6 ind‐yrs	0.006	0.046	−0.030	0.043	No effect
	Woodland	12 ind.; 32 ind‐yrs	0.064	0.333	−0.051	0.179	No effect
Elevation	Riparian	1 ind.; 6 ind‐yrs	2.383^b^	2.590	0.310	4.455	Selection for higher elevation
	Woodland	12 ind.; 32 ind‐yrs	6.274^b^	14.274	1.328	11.219	Selection for higher elevation
Fire frequency	Riparian	1 ind.; 6 ind‐yrs	−0.283^b^	0.056	−0.327	−0.238	Avoidance of frequently burnt areas
	Woodland	12 ind.; 32 ind‐yrs	−0.065	0.205	−0.137	0.006	No effect
Time since last burn	Riparian	1 ind.; 6 ind‐yrs	−0.182	0.382	−0.487	0.123	No effect
	Woodland	12 ind.; 32 ind‐yrs	−0.320^b^	0.582	−0.522	−0.119	Avoidance of long unburnt areas

*Note:* Sample sizes (*n*) represent the number of individuals and individual‐years contributing to each model.

^a^ Terrain Ruggedness Index.

^b^ Statistically significant—CI's do not overlap zero.

Woodland breeders strongly selected *Eucalyptus tetrodonta* woodlands (*β* = 1.770, 95% CI = 1.336 to 2.204), whereas the single tracked riparian breeder strongly avoided them (*β* = −2.500, 95% CI = −2.775 to −2.224). In contrast, the riparian breeder selected areas closer to water (*β* = −0.003, 95% CI = −0.004 to −0.002), whereas woodland breeders showed a slight preference for areas further from water, though the effect size was small but significant (*β* = 0.000, 95% CI = 0.000 to 0.001).

Responses to fire also varied by habitat type. Woodland breeders avoided areas of long unburnt vegetation (i.e., higher TSLB; *β* = −0.320, 95% CI = −0.522 to −0.119), whereas the riparian breeder avoided areas that were frequently burnt (i.e., high fire frequency; *β* = −0.283, 95% CI = −0.327 to −0.238).

**FIGURE 8:**
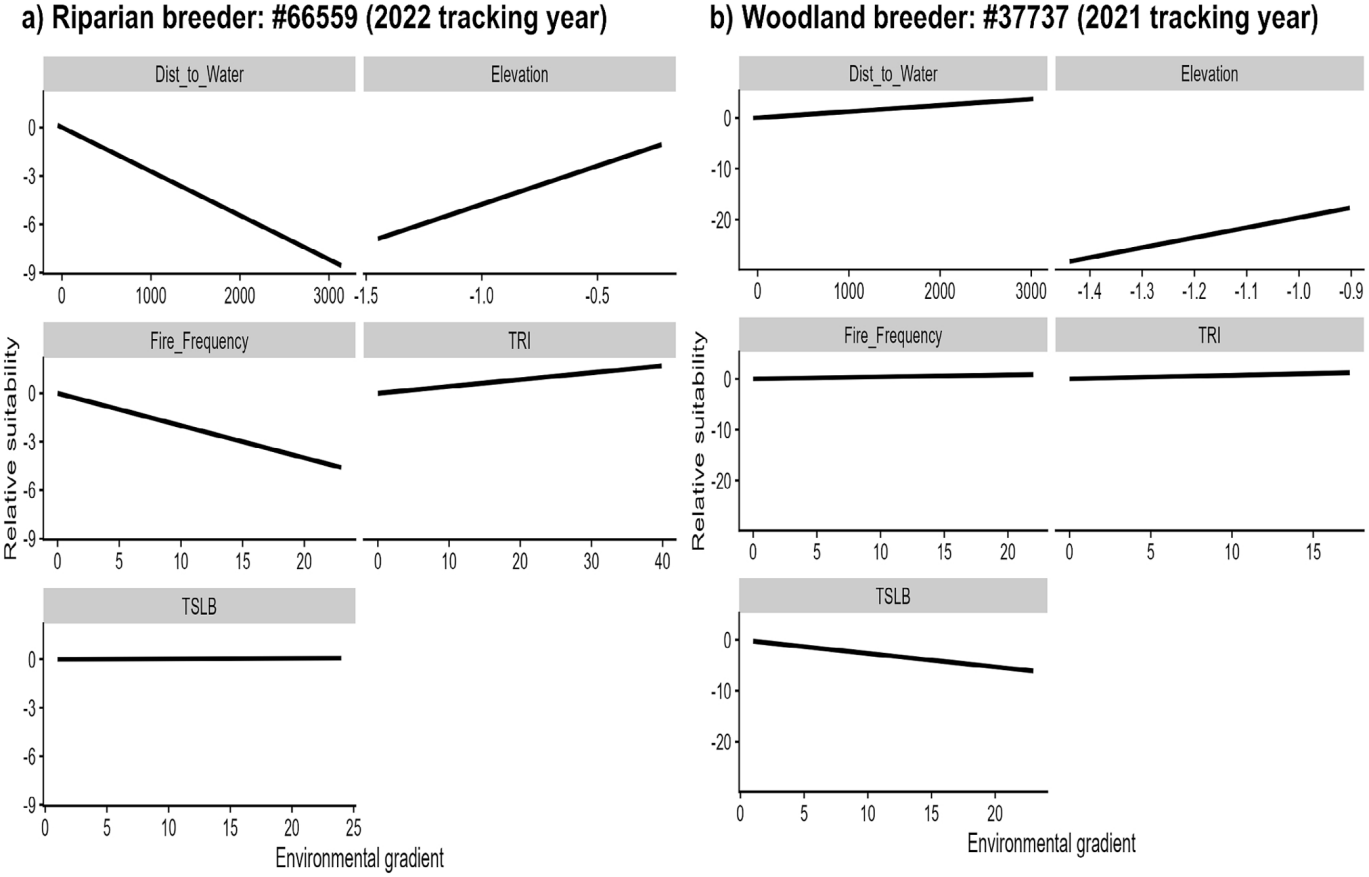
Habitat response curves for representative Red Goshawk individuals occupying (a) riparian and (b) woodland breeding habitats. Only continuous environmental covariates are shown, as binary covariates (e.g., clearing and Tetrodonta woodlands) are not scalable (i.e., values are 0 or 1).

These contrasting selection patterns were also evident in habitat response curves for woodland and riparian individuals (Figure 8). These curves illustrate differences in selection between the two distinct breeding habitats occupied by Red Goshawks across northern Australia. However, the sample size for tracked riparian birds is limited, and these patterns should therefore be considered preliminary and subject to further investigation.

#### 3.2.3 RSF Sensitivity Analysis

Red Goshawks showed no clear group‐level response to cleared areas in mining landscapes (*β* = −0.446, 95% CI = −0.973 to 0.081), despite a negative mean estimate suggesting a tendency toward avoidance and qualitative evidence of spatial separation between movement locations and cleared areas (see Figure 4). In contrast, the signal for restored areas was much clearer, with statistically strong avoidance across all sampled individuals for whom these habitats were available (*β* = −3.233, 95% CI = −5.562 to −0.904).

To assess the influence of the single individual exhibiting selection for cleared areas (#66564), we conducted a sensitivity analysis excluding this individual. This resulted in a stronger negative effect, with confidence intervals no longer overlapping zero (*β* = −0.727, 95% CI = −1.156 to −0.299), indicating statistically significant avoidance of cleared areas across the remaining individuals (*n* = 5 individuals, 13 individual‐years; see Data S6).

**FIGURE 9:**
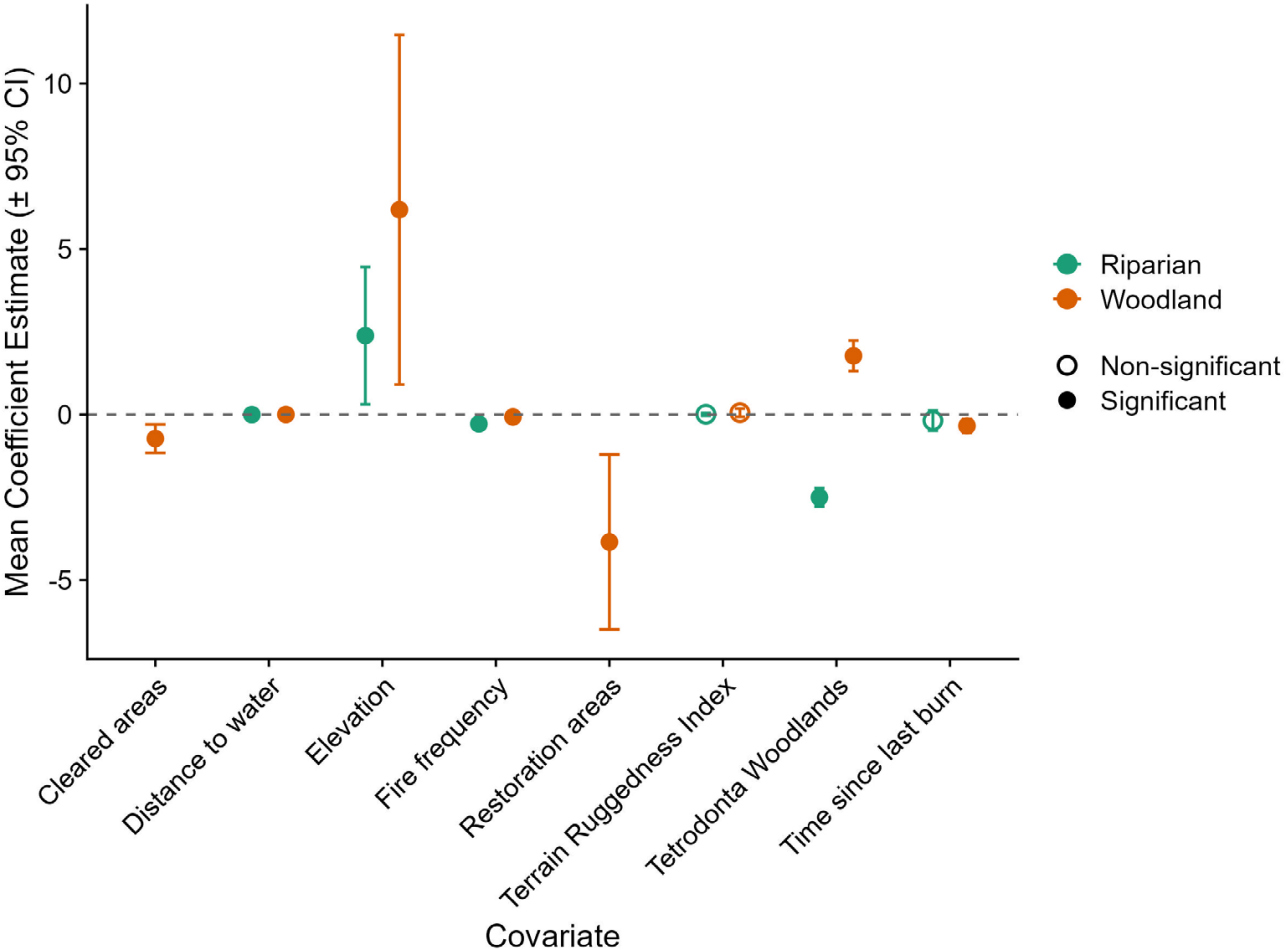
Group‐level RSF coefficient estimates (mean ± 95% CI) by breeding habitat type (woodland and riparian) following sensitivity analysis excluding outlier individual #66564. Statistically significant estimates are indicated by filled circles. Clearing and fire frequency now show statistically significant avoidance following sensitivity analysis compared to the original model (see Table 4).

Estimates for other covariates changed only slightly and did not alter their direction or broad interpretation of habitat selection in this species. However, fire frequency for woodland birds showed a weak negative effect, with confidence intervals narrowly excluding zero (*β* = −0.0746, 95% CI = −0.149 to 0.0002), indicating statistically significant avoidance of frequently burnt areas. Although the effect size was small, this pattern is now consistent with results for the single tracked riparian breeder, indicating broad avoidance of frequently burnt areas by Red Goshawks irrespective of breeding habitat type (Figure 9). However, these results also highlight the sensitivity of grouped responses to individual‐level variation in behaviour.

## 4 Discussion

We quantify for the first time the spatial requirements of Red Goshawks using high resolution movement data, including cumulative and annual home range size, and how these vary by age, breeding status, brood size and habitat type. We also identify habitat selection patterns based on relative use and availability, including individual‐ and group‐level responses to disturbance regimes such as habitat clearing, restoration and fire regime. Together, these results provide new insights into the spatial ecology of this poorly understood species and a foundation for important future research and conservation planning.

However, many of these findings should be considered preliminary, given uneven sampling across key groups within the tracking dataset. In particular, our results are influenced by a strongly female‐biased sample and a skewed representation of individuals occupying woodland breeding habitats relative to riparian systems. Accordingly, inferences relating to these groups should be made with caution. Additional data are required to fully resolve some of the patterns identified in this study in greater detail.

### 4.1 Factors Influencing Space Requirements of Red Goshawk

The substantial home ranges occupied by Red Goshawks reflect global trends among raptors, whereby home range size increases with body mass and the proportion of avian prey in the diet (Peery 2000). Consistent with this relationship, the mean home range size of Red Goshawks ranks among the largest reported in raptors of comparable body size and trophic ecology (Peery 2000), including other large, bird‐eating specialists such as the Peregrine Falcon ( *Falco peregrinus* ) and Gyrfalcon ( *Falco rusticolus* ). Large spatial requirements are characteristic of raptors occurring at naturally low densities (Newton 1979), which can increase sensitivity to environmental change (O'Donnell and del Barco‐Trillo 2020; Broekman et al. 2024). Consequently, individuals occupying large home ranges are likely to be exposed to anthropogenic threats operating across broader spatial scales, reinforcing the need for landscape‐scale conservation action.

Also consistent with global patterns, non‐breeding individuals required substantially larger home ranges compared to breeders with established territories (Tanferna et al. 2013; Braham et al. 2015). This behaviour likely reflects their lack of territory defence and the need to range more widely to access resources and find vacancies in the breeding population (Eisaguirre et al. 2022). Such extensive movements suggest that large, connected landscapes are important to supporting territorial adults as well as non‐breeding individuals transitioning into the breeding population.

Additional variation in home range size was associated with age and annual brood size (Figure 5). Breeding attempts that produced two fledglings were linked to larger home ranges than those producing a single fledgling (Figure 5a). This pattern is consistent with increased foraging demands being placed on adults provisioning multiple offspring. Moreover, previous evidence shows that Red Goshawks are specialist hunters focusing on specific prey species (MacColl et al. 2024), which may require adults to forage over larger areas to provision chicks, particularly if flexibility in prey choice is limited.

In contrast, failed breeding attempts were characterised by the largest home range sizes overall, indicating that individuals released from chick‐rearing duties often expanded their movements beyond the typical breeding territory (Braham et al. 2015). This pattern may also reflect breeding dispersal behaviour and subsequent territory turnover (e.g., Tolvanen et al. 2017; Väli and Rohtla 2025), with tagged females occasionally observed vacating territories following breeding failure, resulting in larger areas utilised during those tracking years.

Individuals tagged as juveniles and subsequently tracked into older age classes (1–4 years old) displayed decreasing home range size each year (Figure 5b). This reduction in space use is likely attributed to these individuals obtaining breeding status (see Table 3) and holding down (and defending) more defined territories (e.g., Kocina and Aagaard 2021; García‐Macía et al. 2024).

### 4.2 Important Habitat Features and Potential Ecological Niche Differentiation

At the home range scale, Red Goshawks consistently selected areas of relatively higher elevation based on what was available, irrespective of breeding habitat type (Figure 6; Table 4). For the single tracked riparian breeder nesting in old‐growth *Melaleuca leucadendra* gallery forest, this pattern may reflect a tendency for these habitats to occur along creeks situated higher in catchment areas. These areas may provide access to spring‐fed permanent water emerging from sloping topography, as well as lower water velocity during run‐off events, which can otherwise bring down trees lower in the catchment and limit the development of tall, old‐growth structure. Similarly, woodland breeders are typically found nesting on relatively elevated topography along low rises and ridgelines, as this is where *E. tetrodonta* woodland communities naturally occur.

Woodland breeders showed strong selection for *E. tetrodonta* woodlands despite the availability of other vegetation communities within their home ranges, indicating that this habitat type meets most of their key ecological requirements. Woodland birds also selected areas marginally further from water, with this effect being statistically significant though small (Table 4). Prior studies suggested that Red Goshawks ‘invariably’ nest within 1 km of permanent fresh water (Aumann and Baker‐Gabb 1991), but our data show woodland nests can be located up to ~2.5 km from water, indicating greater variability in nest placement than previously recognised. This pattern applies specifically to woodland nests given that riparian nests occur within the freshwater ecosystem itself. Creek lines commonly drain from *E. tetrodonta* woodlands which naturally occur on elevated landforms. Selection for areas further from water should be interpreted as a relative effect within these woodland landscapes, rather than a preference for markedly drier habitats. These findings likely reflect the tendency for woodland birds to spend most of their time within the woodland habitat matrix, where access to water remains relatively available across the home range. This appears to be the case within core areas surrounding nests, where a mean core range of 44.5 km is equal to a circular radius of ~3.8 km and would be expected to intersect a watercourse based on observed core range patterns (Table 3).

In contrast, the riparian breeder showed strikingly different habitat selection patterns compared to woodland birds, with strong avoidance of *E. tetrodonta* woodlands despite their wide availability across the home range, and with consistent selection for areas closer to water (Table 4). These contrasting selections also extended to fire responses, with the riparian bird avoiding frequently burnt areas, whereas woodland birds avoided long‐unburnt areas. The riparian breeder also occupied a larger home range compared to woodland birds (Table 3), although this comparison is based on a single individual. Nevertheless, this raises the possibility that habitat requirements influence home range size in this species, with riparian habitat occurring in more spatially limited and dispersed patches along creek corridors, in contrast to broad expanses of woodland habitat. This may potentially require riparian birds to range more widely to access suitable habitats and meet their ecological needs, although more data is needed to verify this pattern.

Together, these contrasting habitat selection patterns between woodland and riparian birds provides emerging evidence of ecological niche differentiation within this species, with each breeding habitat type associated with distinct behaviours and requirements; however, inferences for riparian systems remain preliminary. This interpretation is further supported by the occurrence of breeding populations in landscapes largely devoid of tall *E. tetrodonta* woodlands, such as the Kimberley region of Western Australia, where all recorded nests have been found in *M. leucadendra* riparian gallery forest (e.g., Hill 1910; Aumann and Baker‐Gabb 1991). In contrast, all recorded nests on Cape York Peninsula in Queensland have been found in *E. tetrodonta* woodland habitats, with the exception of a single nest (see Study Area for description), including no *M. leucadendra* gallery forest nests identified in our dataset or the historical literature for this region (e.g., Favaloro 1981; Debus et al. 1993). However, we note a possible exception involving a nest reportedly observed in a paperbark tree (*Melaleuca* sp.) along the Mitchell River near Mt. Molloy, Queensland, during the 1980s–1990s (L. Nielsen pers. comm.).

Such ecological differentiation may contribute to regional variation in population size and distribution of Red Goshawk, given that woodland and riparian habitats differ markedly in their extent and availability across northern Australia. While further studies are required, the contrasting spatial configuration of these habitats suggests that home range size and subsequent breeding densities and regional populations may vary between woodland‐dominated regions (e.g., Cape York Peninsula, Tiwi Islands) and riparian‐dominated regions (e.g., the Kimberley, Gulf of Carpentaria, inland Northern Territory).

These differences are likely to have important conservation implications. In woodland systems, where breeding habitat is comparatively more extensive and which probably support higher population densities given apparently smaller home range sizes (Table 3), management may benefit from maintaining large, connected landscapes where broad‐scale habitat loss or degradation are minimised (e.g., via logging, mining, or agricultural development) given the selection for these woodland habitats and avoidance of cleared areas (Table 4; Figure 9). In contrast, riparian systems are geographically widespread but contain only limited patches of suitable breeding habitat ( *M. leucadendra* gallery forest), often in less productive inland areas, and probably support lower breeding densities (Table 3). These populations may therefore be more sensitive to processes that further reduce habitat availability, such as loss of old‐growth riparian tree stands, fire encroachment into riparian zones, or reduced water availability (e.g., by extraction or climate variability). Accordingly, conservation strategies are likely to be more effective if they account for regional differences in habitat structure and associated threat processes, including differing approaches to land management and threat mitigation (Yong et al. 2023).

### 4.3 Implications for Landscape Fire Management

Our results show contrasting fire responses between breeding habitat types across Australia's tropical savannas. Woodland birds avoided long‐unburnt areas, consistent with a preference for the semi‐open woodland structure maintained by regular, low‐intensity burning. In contrast, the riparian breeder avoided frequently burnt areas and remained close to water, highlighting the potential impact of fire intrusion into riparian gallery forests used by Red Goshawks. Indeed, such fires pose a direct threat to nests, particularly late in the dry season (Sept‐Oct) when chicks are still confined to nests, while also contributing to the long‐term degradation of large tree communities. Nest trees have even been observed burning within riparian habitats (Data S7), and there are also documented cases of nests being incinerated with chicks inside them (Hogarth 1991; L. Cupper pers. comm.).

Inappropriate fire regimes are a known threatening process to biodiversity throughout Australia's tropical savannas and could therefore be a key threatening process impacting the Red Goshawk (Woinarski and Legge 2013). Inappropriate fire regimes can alter savanna vegetation structure in several ways, with too‐frequent late‐season fires reducing mature trees and understorey complexity necessary for some species to survive and reproduce (Murphy and Legge 2007; Radford et al. 2015; Teunissen et al. 2023). Conversely, insufficient fire can lead to woody thickening, which can be detrimental to species requiring a level of habitat openness, such as the grassland‐specialist Golden‐shouldered Parrot (*Psephotellus chrysopterygius*) (Crowley et al. 2025) and Red Goshawks occupying semi‐open woodlands.

The fire‐selection patterns detected here suggest that fire management for Red Goshawks needs to be habitat‐specific: maintaining patchy, low‐intensity fire in woodlands while avoiding fire intrusion into riparian systems. However, we note that these patterns are based on low sample size in riparian systems, and that further research is required to resolve these relationships more clearly. Broader issues such as invasive grasses (i.e., Gamba Grass; *Andropogon gayanus* ) and grazing impacts can further alter fire behaviour and habitat structure in these systems (Radford et al. 2015; Rossiter‐Rachor et al. 2023), but evaluating these drivers was outside the scope of this study and will require targeted future work.

### 4.4 Use of Cleared and Restored Habitats

In mining landscapes on western Cape York Peninsula, both cleared areas and restored habitats were avoided by Red Goshawks (Table 4; Figure 9). The extent to which restored areas may function as suitable habitat remains uncertain based on present conditions. Although most tracked individuals (*n* = 4/6) were exposed only to early‐stage restoration areas (see Figure 4), the others for whom more mature restored habitat was available in their home range also showed avoidance or no measurable response (see Figure 6).

One individual (#66564) did use cleared edges and was occasionally recorded in restored areas, providing the basis for important future research into habitat development within these known‐use patches. However, based on broader selection patterns, any long‐term benefits of habitat restoration remain uncertain and are likely dependent on the structural and floristic features of original *E. tetrodonta* woodlands being successfully replicated. These features include tall, semi‐open canopies with horizontally branched trees dominated by *E. tetrodonta*, Melville Island Bloodwood ( *C. nesophila* ), Cooktown Ironwood (*Erythrophleum chlorostachys*) and, where geographically appropriate (i.e., Northern Territory and Western Australia), Darwin Woollybutt (*Eucalyptus miniata*). Accordingly, restoration efforts should prioritise the retention and recruitment of large trees and the re‐establishment of structurally complex woodland communities that reflect these characteristics.

The avoidance of cleared and structurally simplified habitats indicates that the removal of intact *E. tetrodonta* woodlands directly reduces the amount of usable habitat within Red Goshawk home ranges. These findings provide insight into a potential mechanism by which habitat loss may have contributed to population declines across the species' south‐eastern range (e.g., northern New South Wales, southern Queensland; Seaton 2014; MacColl et al. 2023). Given the observed avoidance of cleared areas (Table 4; Figure 9), and extensive historical clearing of suitable habitat within these regions (Ward et al. 2019), deforestation may have constrained habitat availability and space use for resident Red Goshawks. The species' subsequent extirpation from these regions occurred prior to any targeted research or management efforts aimed at understanding or arresting the decline. Our findings from northern Australia highlight a potential pathway by which habitat loss impacts the species, particularly within the more coastal and wooded parts of the species' range.

### 4.5 Protection Buffers

Breeding Red Goshawks consistently relied on well‐defined core ranges (50% wAKDE) centred on nest sites, with movements concentrated within an average area of 44.5 km^2^ (Table 3; Figure 2b). These core ranges represent the areas of highest use, accounting for 50% of the utilisation distribution. However, throughout the year breeding adults used much larger areas (mean 95% wAKDE = 411.9 km2; Table 3; Figure 2b), particularly outside the breeding season (~Jan‐April). This shows that although Red Goshawks occupy expansive areas, their core ranges represent only ~10% of overall home ranges, highlighting how strongly space‐use is tied to the nesting area.

Existing government policy on nest‐protection buffer zones ranges from 200 to 300 m around nests, representing an area of just 0.13–0.28 km2. These buffers therefore protect < 1% of core areas and an even smaller proportion of overall home ranges (Table 3). Such buffers are often applied in disturbed landscapes, such as those associated with native forest logging, plantation forestry and mining operations, and appear designed primarily to prevent direct disturbance to the nest tree and its immediate surroundings, rather than to sustain breeding territories over the long term.

While these buffers do not encompass the full extent of core ranges, there is some evidence to suggest that nest occupancy and successful breeding can be maintained when buffers (larger than 200–300 m) are combined with connectivity to undisturbed habitat. The examples referred to here are those nests depicted in year‐to‐year clearing analysis (Figure 4), which illustrates their spatial context relative to surrounding habitat clearance. This suggests that Red Goshawks can tolerate some degree of local disturbance provided sufficient habitat is retained within the core range while providing access to larger, intact areas across the broader home range extent. However, the disturbance threshold beyond which occupancy or breeding success declines remains unknown and warrants further investigation as a key research priority identified in the species recovery plan (Queensland Department of Environment and Resource Management 2012). In the absence of such evidence, our results support a precautionary approach that gives priority to habitat retention and connectivity within core breeding areas.

More broadly, our findings indicate that conservation policy aimed at protecting Red Goshawk populations should consider habitat retention at spatial scales far larger than those currently applied in regulatory buffers. Increasing the representation of Red Goshawk habitats—such as *E. tetrodonta* woodlands—within Australia's protected area estate may improve the species' long‐term trajectory. Ultimately, however, policies which better account for the exceptionally large spatial requirements of Red Goshawks are necessary to safeguard the species, particularly given the growing land‐use pressure being placed on its habitats.

### 4.6 Future Research

The distinct spatial requirements observed between woodland and riparian Red Goshawks warrant further investigation as our research indicates these habitats represent unique ecological niches occupied by the species across its tropical savanna breeding range. The riparian niche ultimately extends the species' distribution further inland beyond predominantly near‐coastal woodland communities, including all known breeding territories in the Kimberley region of Western Australia, and potentially including the Pilbara region where adult‐age birds have now been verified (MacColl et al. 2023). Improving our understanding of the ecology of Red Goshawks in these habitats will greatly improve range‐wide conservation and recovery planning.

The other notably under‐sampled group in this study was adult males, with data obtained only from a single individual. Males fulfil a critical role in nest provisioning and territory maintenance, and as such, an understanding of their movements has important implications for management and conservation planning. However, tracking durations were consistently lower in tagged males (see Table 1), and any future tagging efforts should strongly consider potential impacts and assess whether alternative tagging or study methodologies are needed.

The verified occurrence of a single Red Goshawk recorded occasionally in restoration areas presents a valuable opportunity to investigate the structural attributes that may eventually make restored habitats become suitable for this species. Assessing these conditions alongside other ecological requirements, such as prey availability, would provide an important case study to guide the design and evaluation of any future restoration programs.

In addition, future research should investigate how Red Goshawks use modified landscapes in greater spatial and temporal detail than examined in the present study and also to determine the disturbance thresholds beyond which nest occupancy or breeding success declines. Such knowledge is essential for informing adaptive management approaches that ensure buffer designs and surrounding landscape management remain effective in supporting conservation of the species within disturbed landscapes.

## 5 Conclusion

Our research shows that the persistence of Red Goshawks across northern Australia depends on maintaining extensive tracts of wooded and well‐watered habitats. Tall, structurally complex woodlands—such as Darwin Stringybark (*Eucalyptus tetrodonta*) on ridges and rises, and Weeping Paperbark ( *Melaleuca leucadendra* ) gallery forest on freshwater creek systems—represent important habitats supporting breeding territories. Importantly, our results reveal some evidence of ecological niche differentiation, with woodland and riparian breeders exhibiting distinct habitat preferences, fire responses and spatial requirements. This differentiation may help explain the species' distribution across contrasting landscapes, from well‐wooded near‐coastal areas to more open savanna ecosystems further inland where riparian habitats are critical refugia.

This species' exceptionally low population densities, combined with its requirement for extensive areas of intact habitat, suggest that habitat clearing and degradation are likely to undermine breeding territories and place additional pressure on already small, persisting populations. Targeted fire management offers a practical tool for sustaining these territories, with regular, low‐intensity burning needed to maintain semi‐open woodland structure, and fire exclusion essential in old‐growth riparian habitats. Furthermore, improved habitat protection protocols across known breeding territories and core areas are important to consider, especially where land use activities could reduce habitat availability or condition.

## Author Contributions

**Christopher MacColl:** conceptualization (lead), data curation (lead), formal analysis (lead), funding acquisition (lead), investigation (lead), methodology (equal), project administration (lead), validation (lead), visualization (lead), writing – original draft (lead), writing – review and editing (equal). **Richard Seaton:** conceptualization (equal), data curation (equal), funding acquisition (equal), investigation (equal), methodology (equal), project administration (supporting), resources (supporting), supervision (equal), writing – review and editing (equal). **David A. Stewart:** conceptualization (supporting), data curation (equal), funding acquisition (supporting), investigation (equal), project administration (supporting), resources (supporting), writing – review and editing (supporting). **Nicholas P. Leseberg:** conceptualization (equal), investigation (equal), supervision (equal), validation (equal), writing – review and editing (equal). **Stephen A. Murphy:** conceptualization (equal), investigation (equal), supervision (equal), writing – review and editing (equal). **James E. M. Watson:** conceptualization (equal), investigation (equal), methodology (supporting), project administration (supporting), supervision (lead), writing – review and editing (equal).

## Funding

Funding for this research was provided by Rio Tinto Weipa, with in‐kind support from the Australian Wildlife Conservancy and the Queensland Department of Environment, Tourism, Science, and Innovation. All partner organisations contributed to aspects of study design and required approval of the manuscript for publication; however, they had no role in data analysis, interpretation of results, or preparation of the manuscript.

## Ethics Statement

This research was conducted under the approved conditions of the Queensland Department of Agriculture and Forestry *Animal Ethics Permit* CA 2020/11/1437 (2018–2022) and CA 2023‐05‐1722 (2023), the Queensland *Scientific Purposes Permit* WA0008989 (2019–2023) and WA0053688 (2023), the Queensland National Parks *Permit to Take, Use, Keep or Interfere with Natural Resources* PTU18‐001174 (2018–2021) and PPTUKI‐100107479 (2022–2023), the Northern Territory National Parks *Permit to Interfere with Wildlife for Commercial Purposes* 65,181, the Northern Territory *Licence for Research Involving Animals* 101, the Australian Government *Part 13 Permit* E2019‐0163, and the West Australian *Authorization to Take or Disturb Threatened Species* TFA 2019‐0079.

## Conflicts of Interest

Research on the Red Goshawk was conducted as a collaborative project between the University of Queensland, Rio Tinto Weipa, the Australian Wildlife Conservancy, and the Queensland Department of Environment, Tourism, Science, and Innovation. Funding for this research was provided by Rio Tinto Weipa to support fieldwork costs. C.M. is employed as project manager for this project. All other authors declare no competing interests.

## Supporting information


**Data S1:** (.pdf) Semi‐variograms and utilisation distributions (UDs) for all Red Goshawk individuals used to estimate home range (wAKDE). Each page shows (a) the individual semi‐variogram used to assess range residency and model fit, and (b) the corresponding utilisation distribution, including 95% and 50% isopleths.

**Data S2:** (.csv) Cumulative home range estimates (50% and 95% wAKDE) by individual.

**Data S3:** (.csv) Annual home range estimates (50% and 95% wAKDE) by individual‐year.

**Data S4:** (.csv) Individual‐level RSF coefficient estimates and confidence intervals (all individuals).

**Data S5:** (.csv) Group‐level RSF coefficient estimates and confidence intervals prior to sensitivity analysis (all individuals).

**Data S6:** (.csv) Group‐level RSF coefficient estimates and confidence intervals following sensitivity analysis (excludes individual #66564).

**Data S7:** (.jpg) Photographs of an active Red Goshawk nest tree in riparian habitat before and after fire during the 2021 breeding season. The fire scar extended to the lower fork supporting the tree crown containing the nest and two young; photographs were taken from different angles.

## Data Availability

The raw GPS movement data generated and analysed during this study are not publicly available due to intellectual property restrictions associated with third‐party agreements with industry partners. However, the raw GPS movement dataset is provided as supporting information S1 for review purposes only, not for publication. However, datasets derived from the raw movement data, including individual‐level annual and cumulative home range estimates and habitat selection results, are provided as Supporting Information S1 for publication. Access to raw movement data may be considered upon reasonable request, subject to approval by relevant data custodians and third‐party partners.
