# Supplementary material for: Home Range and Habitat Selection of the Endangered Red Goshawk ( Erythrotriorchis radiatus ) Across Australia's Tropical Savannas
Source: Ecol Evol. 2026 Aug 4;16(8):e74042. doi: 10.1002/ece3.74042 (PMC13435263; doi:10.1002/ece3.74042)

Individual: #202346 – Breeding female (2021 - 2025)

#202346

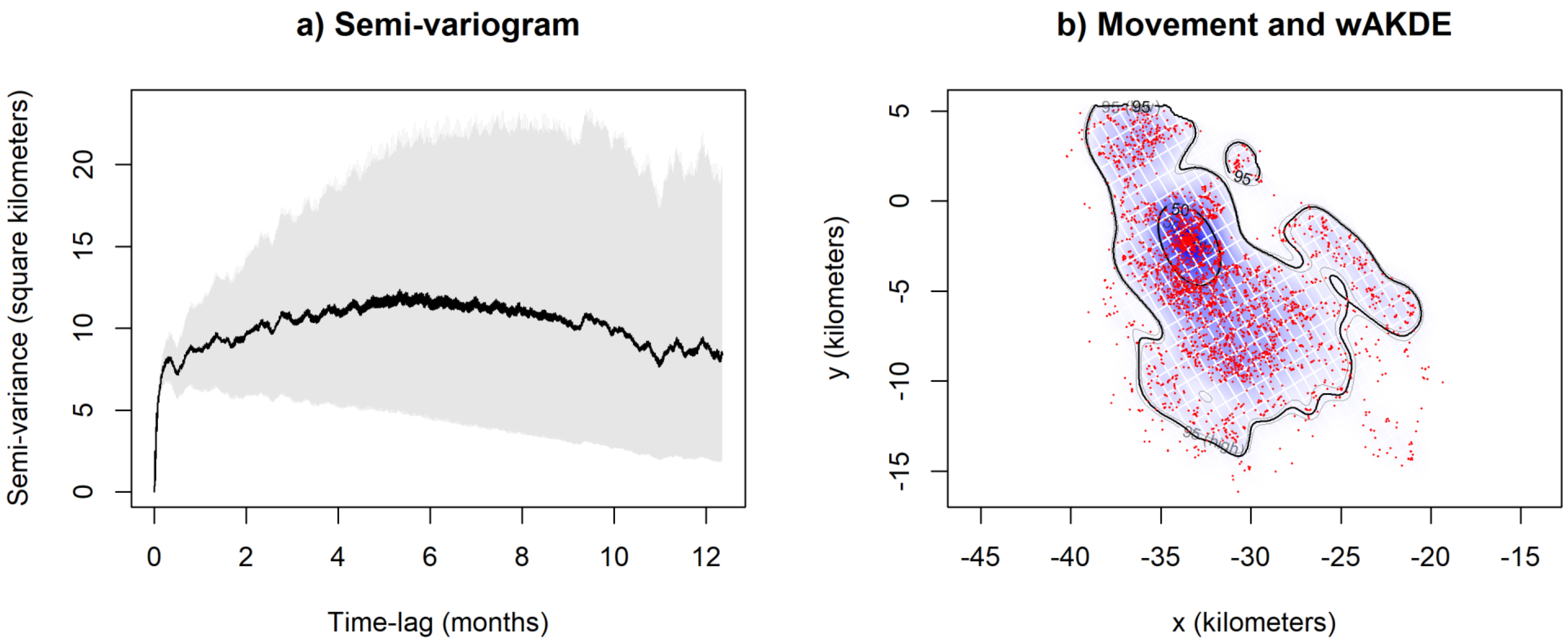

Individual: #220636 – Non-breeding female (2021 - 2024)

#220636

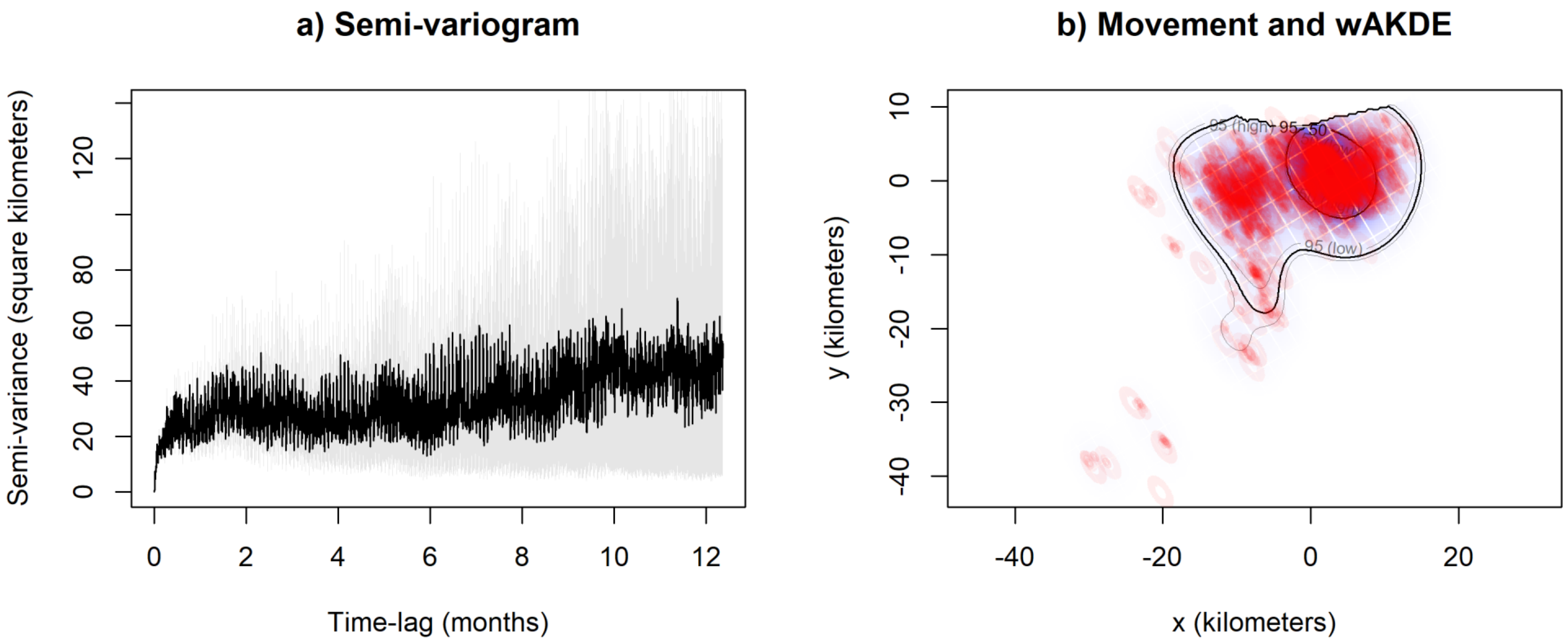

Individual: #37740_2 – Breeding female (2020 - 2024)

#37740

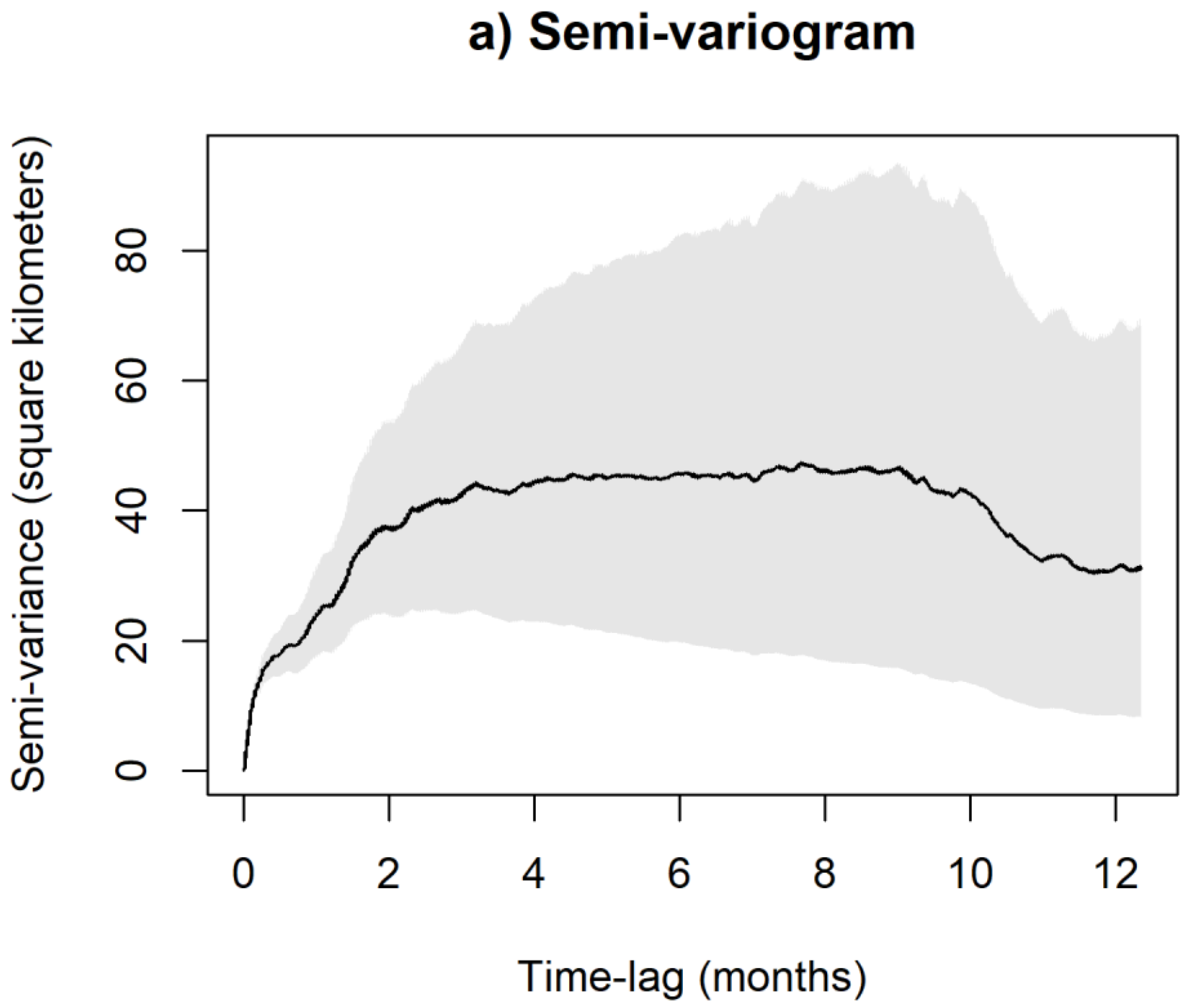

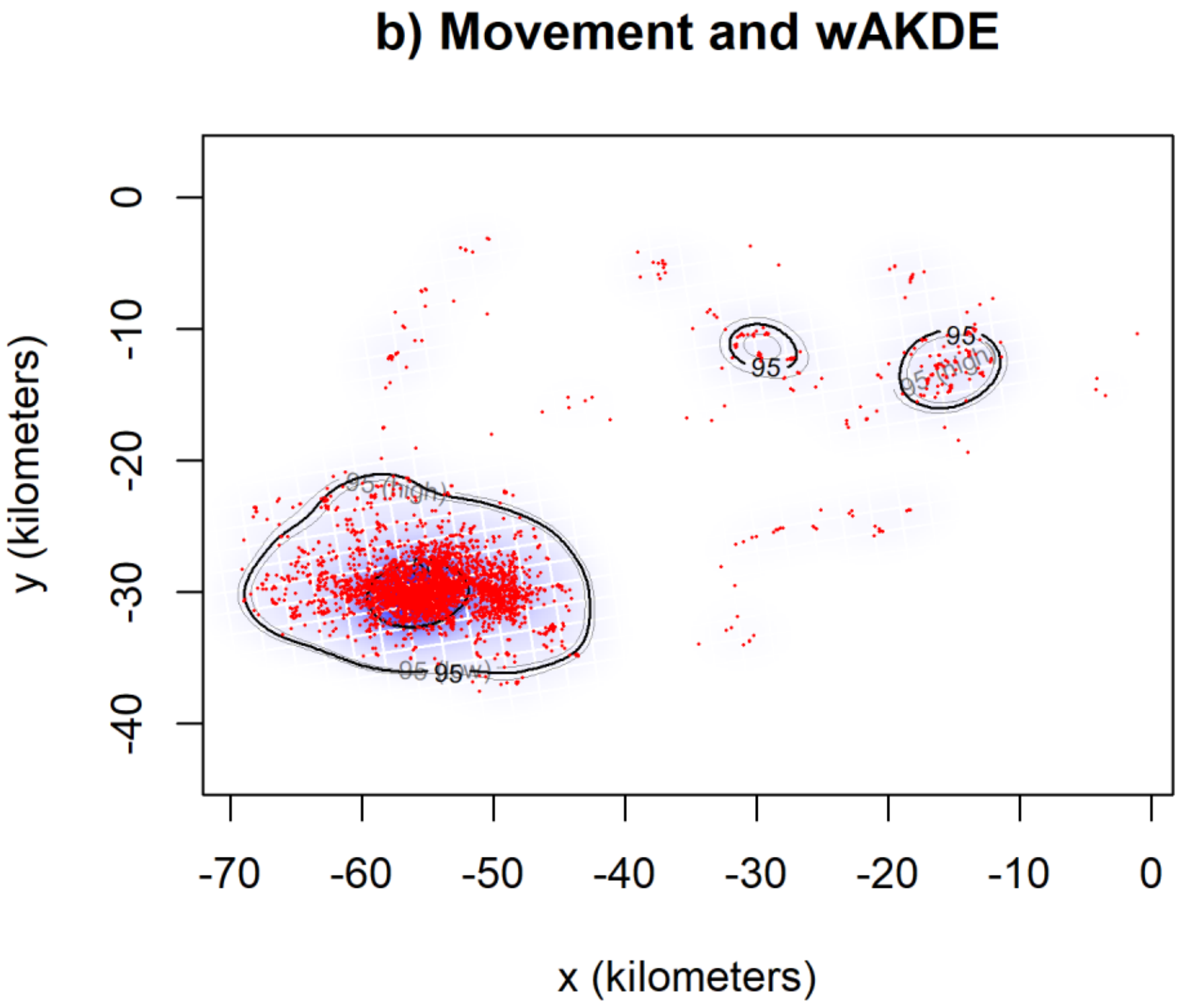

Individual: #66560 – Breeding female (2019 - 2024)

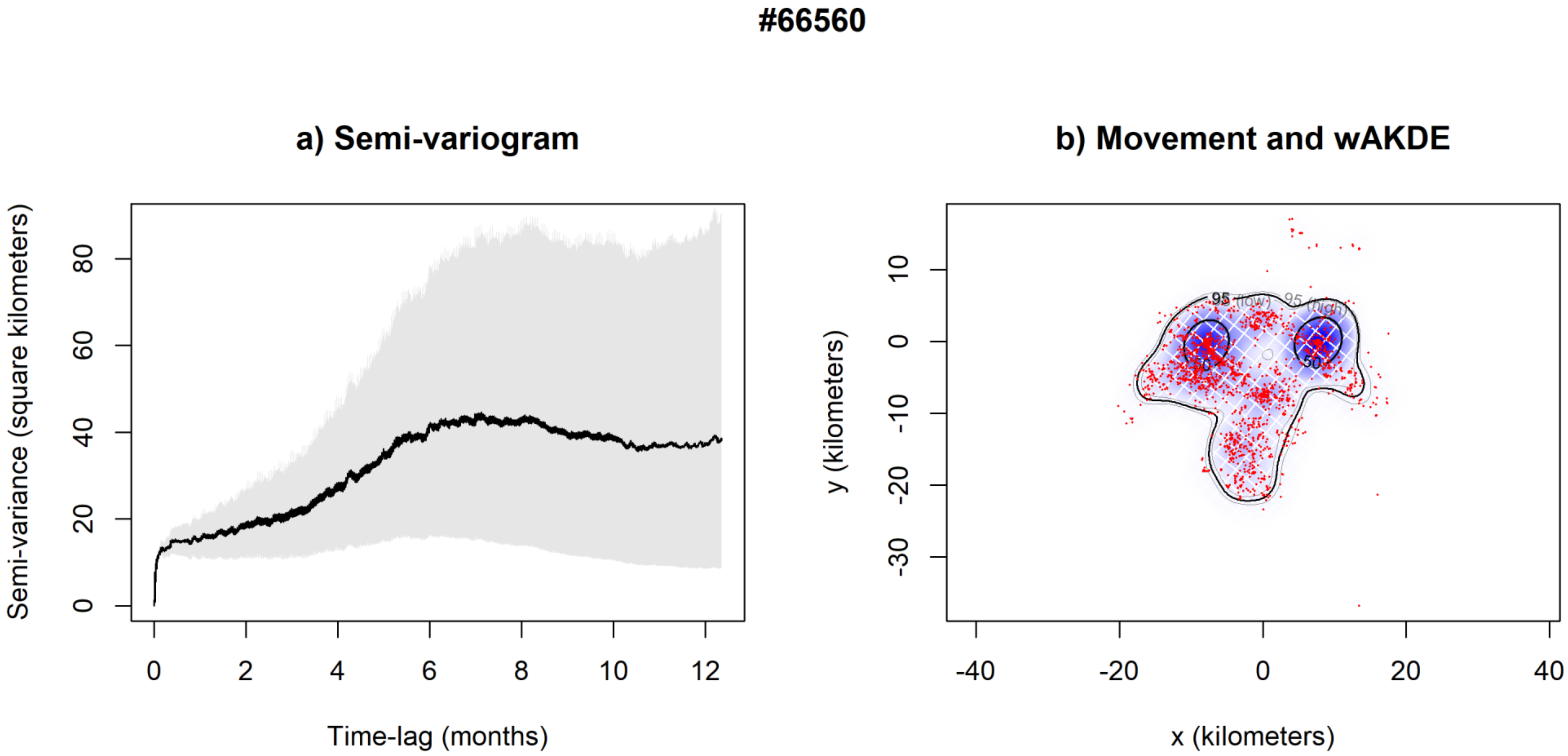

Individual: #226138 – Breeding male (2022)

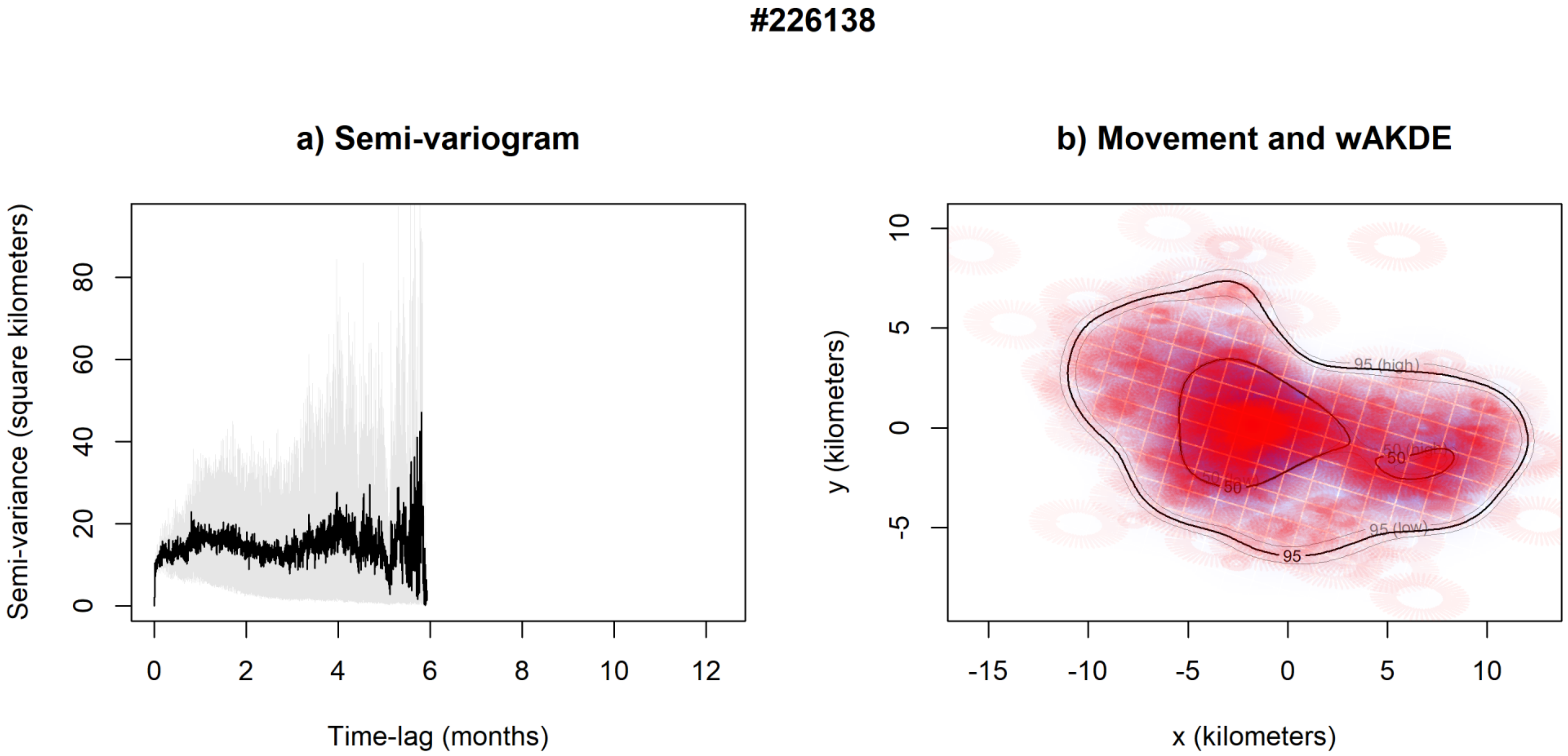

Individual: #157650 – Breeding female (2018 - 2021)

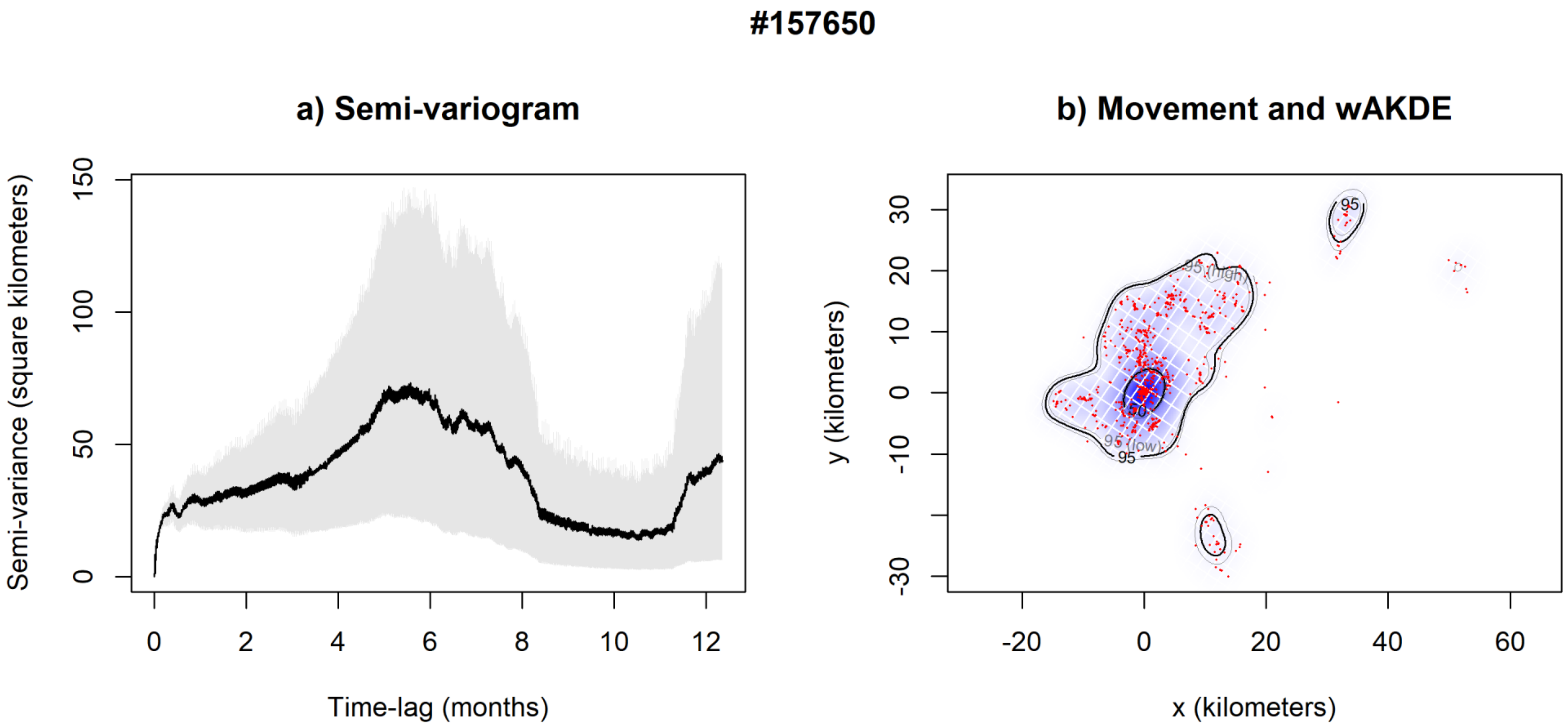

Individual: #157651 – Non-breeding female (2018 - 2019)

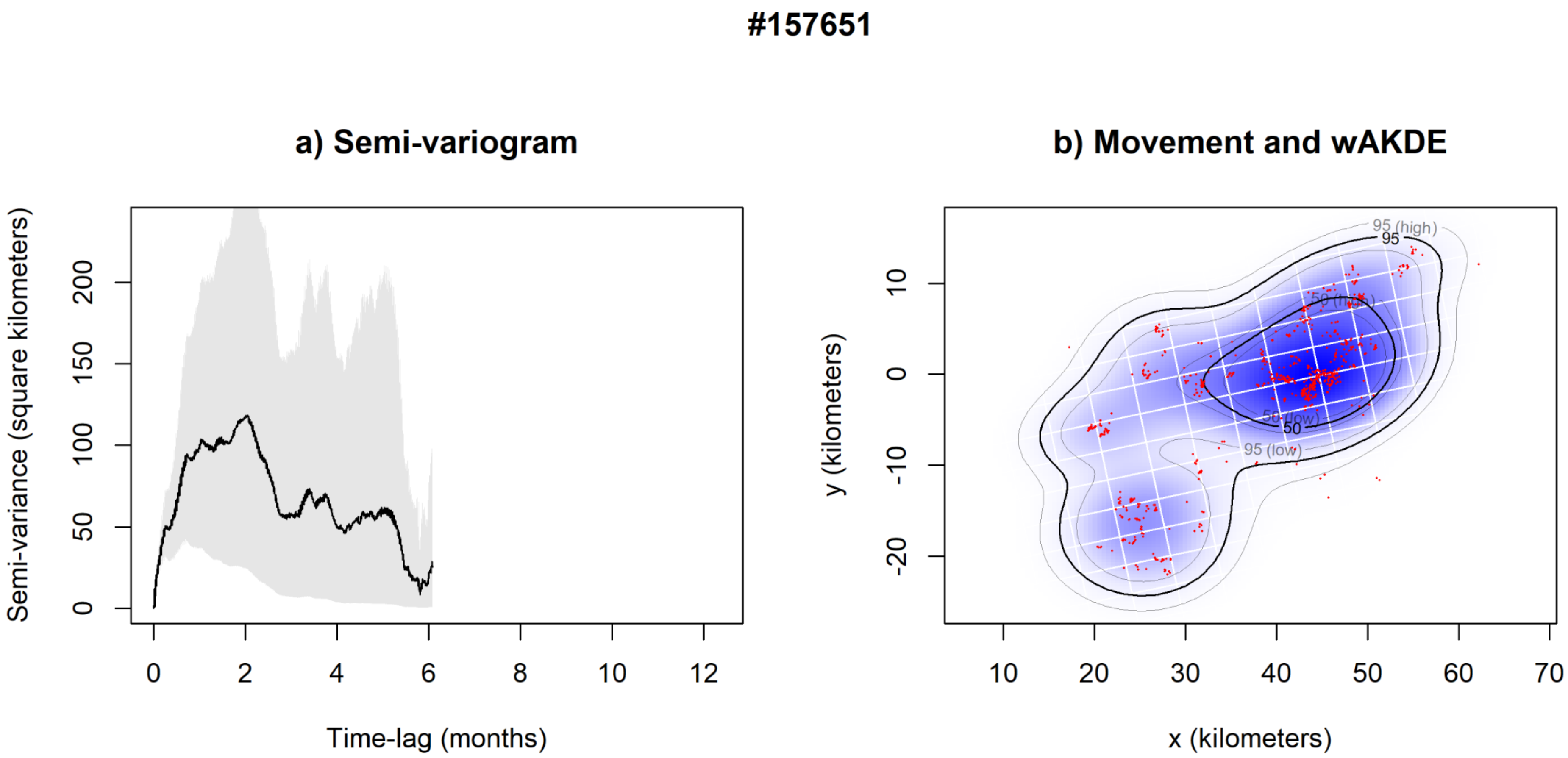

Individual: #150621 – Non-breeding female (2016 - 2017)

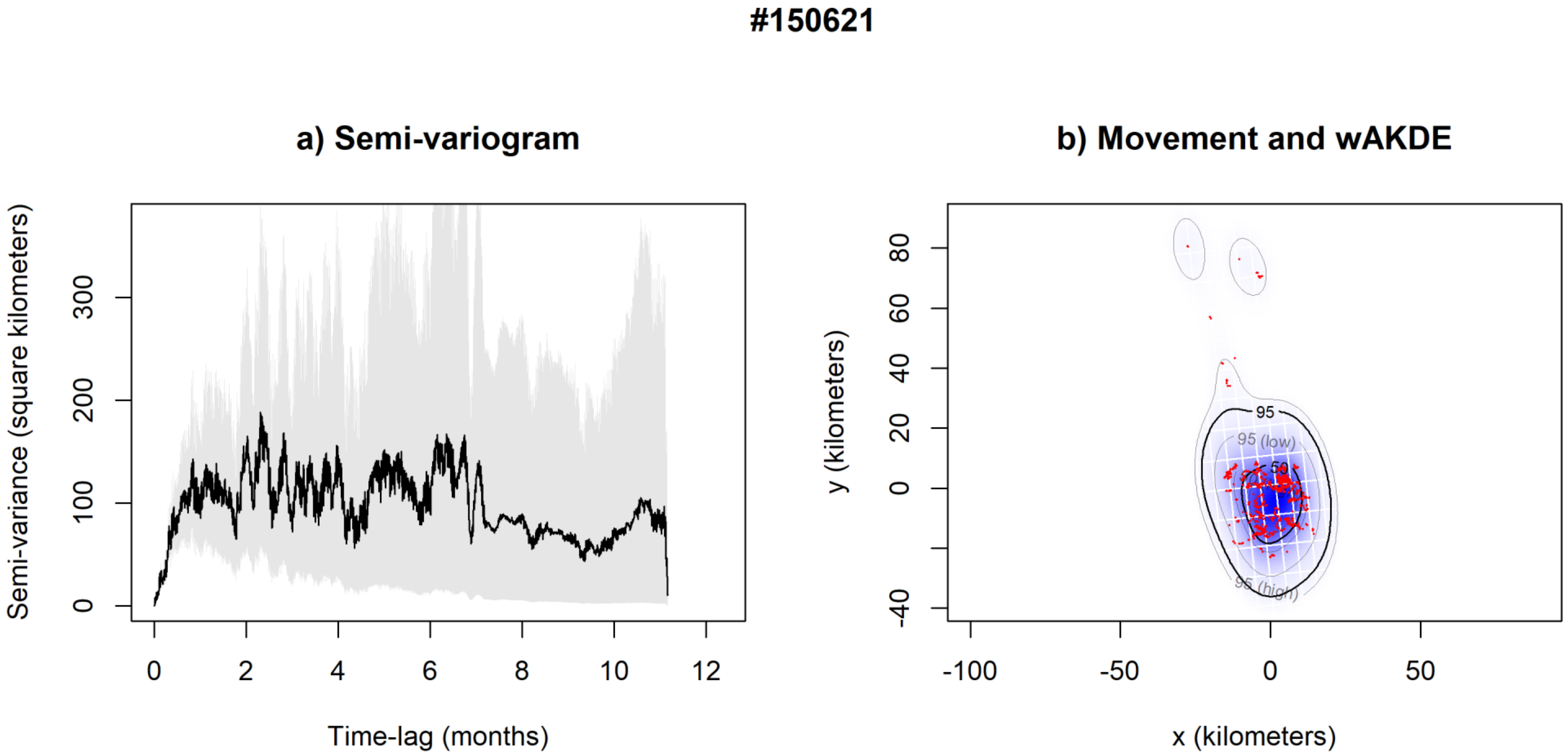

Individual: #37737 – Breeding female (2020 - 2023)

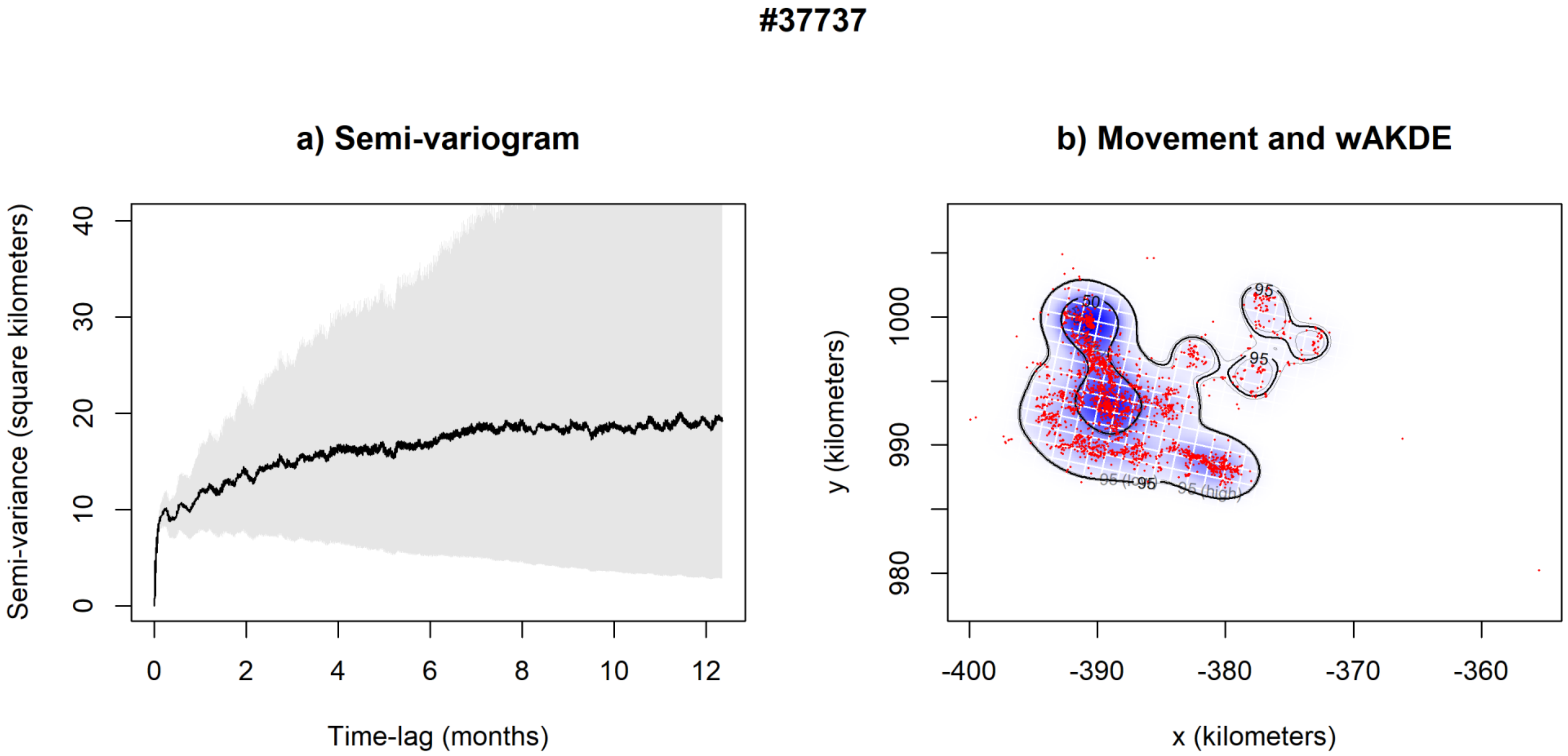

Individual: #66569 – Breeding female (2019 - 2025)

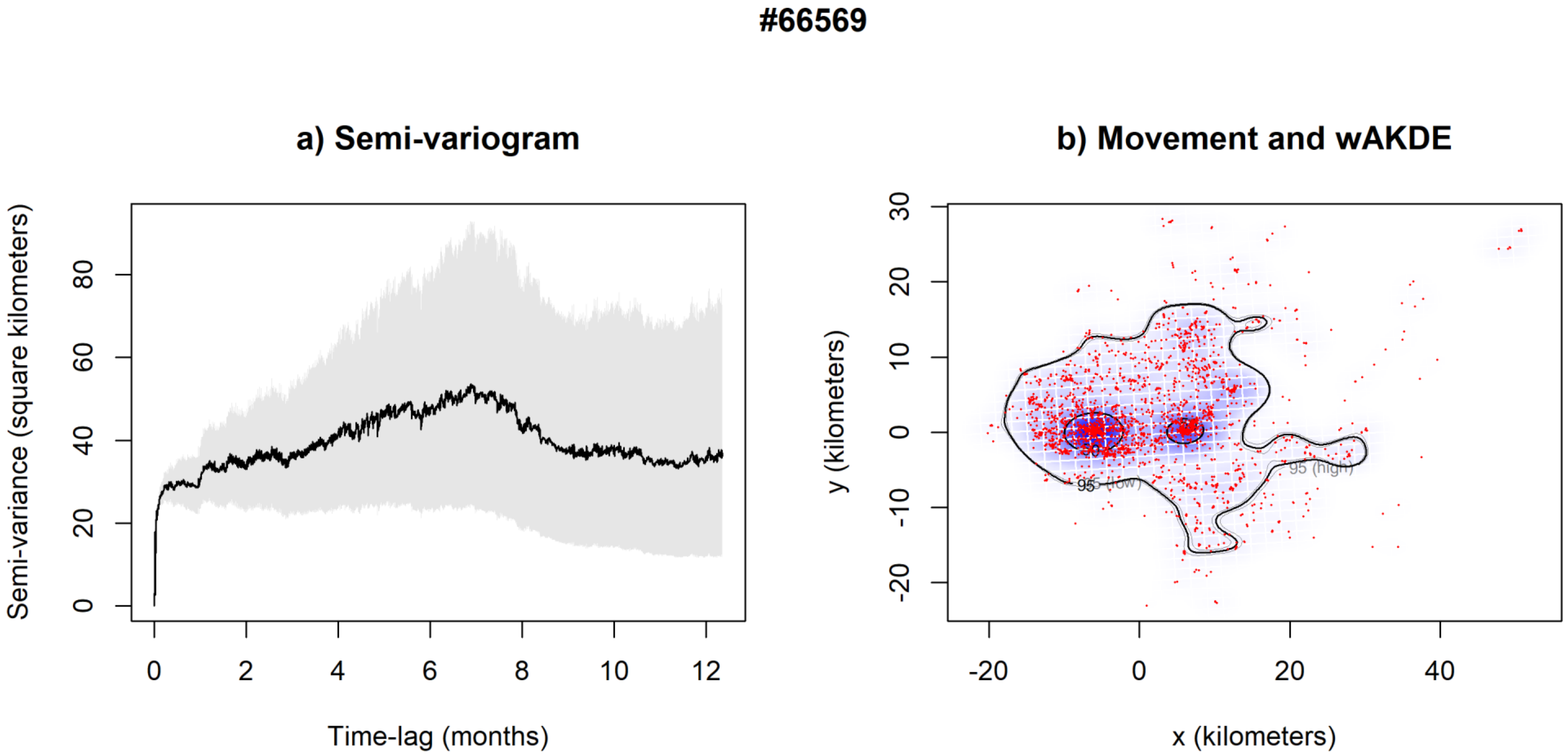

Individual: #202348 – Breeding female (2021 - 2024)

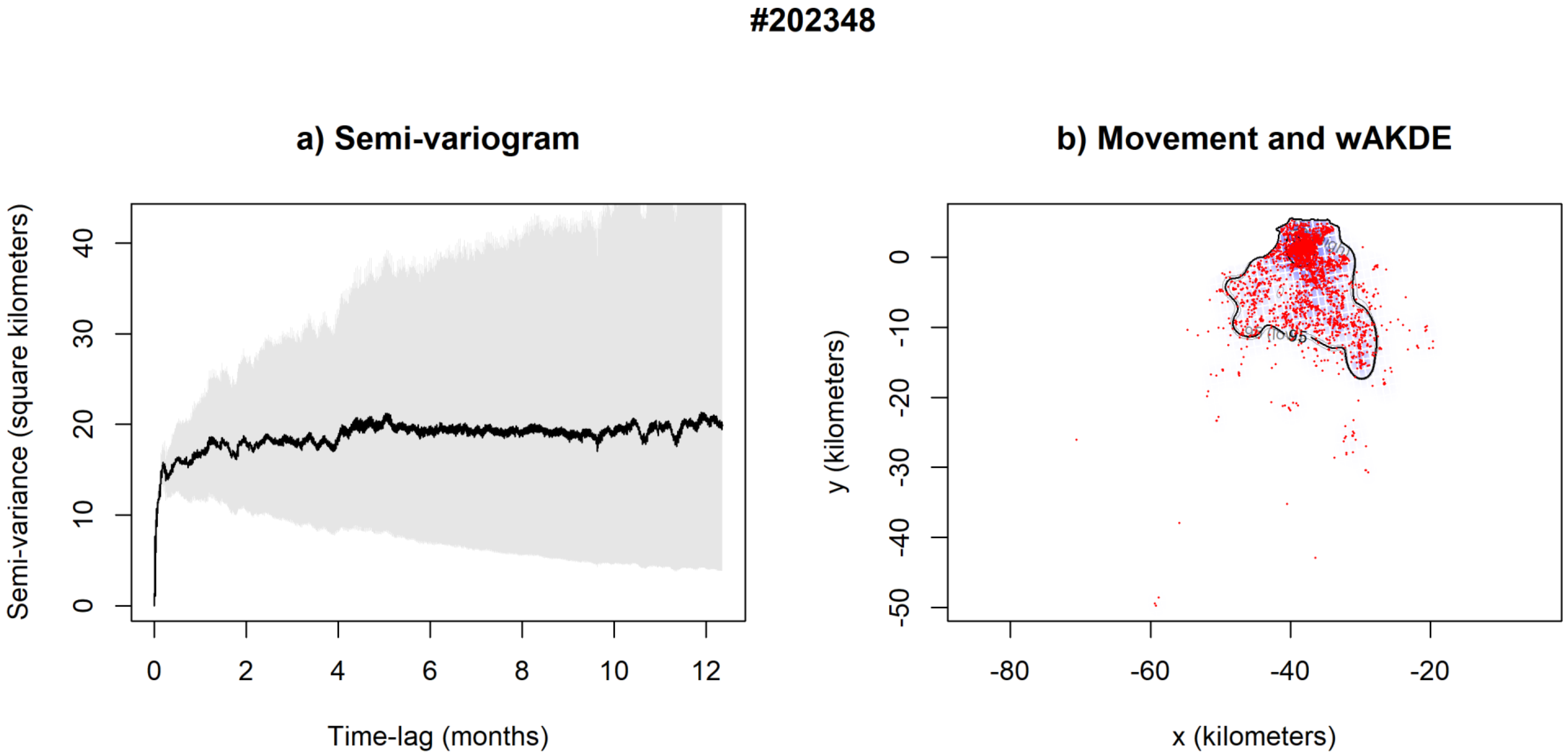

Individual: #66564_2 – Breeding female (2021 - 2024)

#66564

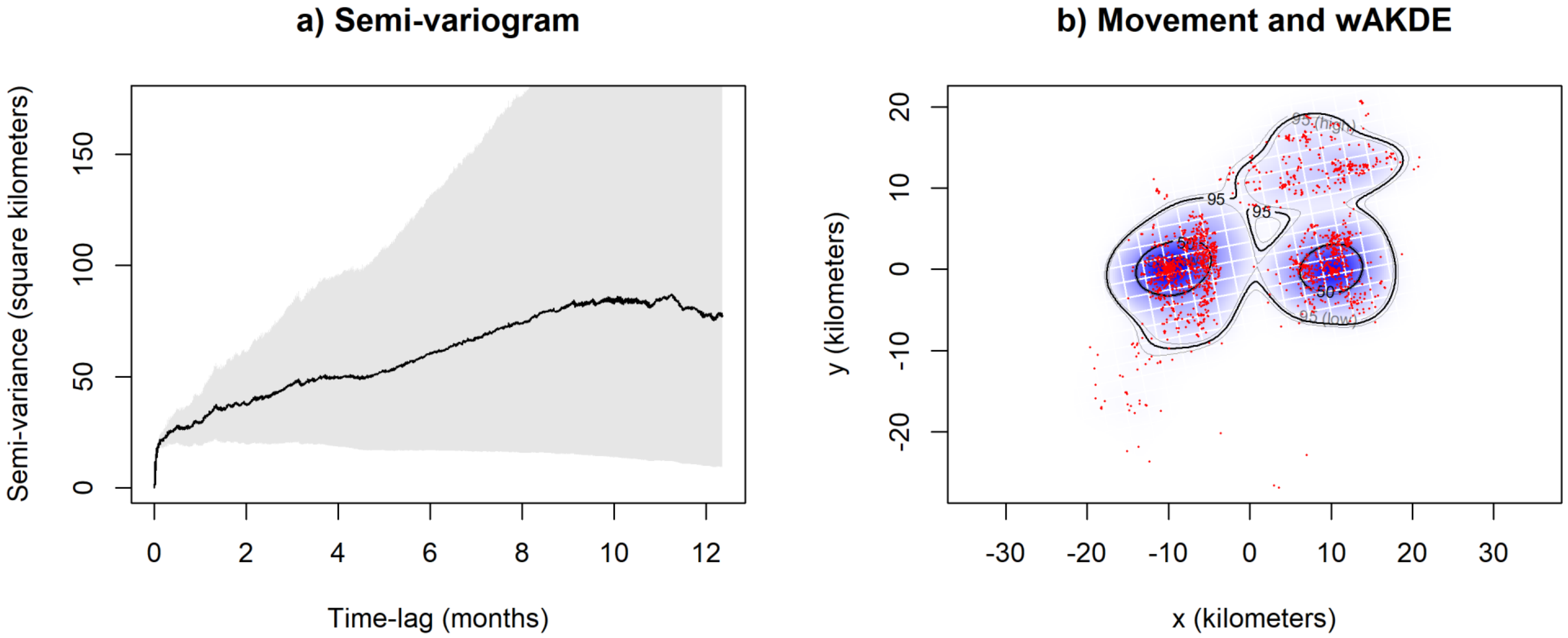

Supplement: Supplementary file 1 [file ECE3-16-e74042-s001.pdf]
